# Differentiation of Laboratory-Obtained *Ixodes ricinus* × *Ixodes persulcatus* Hybrid Ticks: Selection of Suitable Genes

**DOI:** 10.3390/microorganisms10071306

**Published:** 2022-06-27

**Authors:** Alexander G. Litov, Oxana A. Belova, Sergey V. Bugmyrin, Ivan S. Kholodilov, Lidia Iu. Romanova, Galina G. Karganova

**Affiliations:** 1 Chumakov Federal Scientific Center for Research and Development of Immune-and-Biological Products of RAS (Institute of Poliomyelitis), 108819 Moscow, Russia; novosti-wxo@yandex.ru (A.G.L.); mikasusha@bk.ru (O.A.B.); ivan-kholodilov@bk.ru (I.S.K.); lromanova1@bk.ru (L.I.R.); 2 Institute of Biology, Karelian Research Centre, Russian Academy of Sciences, 185910 Petrozavodsk, Russia; sbugmyr@mail.ru; 3 Department of Organization and Technology of Immunobiological Preparations, Institute for Translational Medicine and Biotechnology, Sechenov First Moscow State Medical University, 119991 Moscow, Russia; 4 Department of Virology, Faculty of Biology, Lomonosov Moscow State University, 119234 Moscow, Russia

**Keywords:** *Ixodes persulcatus*, *Ixodes ricinus*, hybrid, ITS, Toll, sex-linked inheritance, rRNA, lysozyme

## Abstract

*Ixodes ricinus* and *Ixodes persulcatus* ticks are the main vectors of tick-borne encephalitis virus and some bacterial pathogens. The regions where these tick species live overlap, forming large sympatric areas. It has previously been shown that these tick species have no morphological barrier, and interspecies crossing is possible with the appearance of sterile hybrids. It has also been shown that hybrid larvae and nymphs can be differentiated using discriminant functions based on a set of morphological features. However, such an approach is laborious and rather ineffective with adult ticks, making a molecular approach necessary. In the current work, we tested the ability of different systems to differentiate laboratory-obtained hybrid ticks. Our data suggest that commonly used primer sets that target rRNA are unsuitable for hybrid tick determination, likely due to the rRNA region being linked with the X chromosome in *I. ricinus* and *I. persulcatus* ticks. We tested several primer sets targeting different non rRNA genes to assess their ability to determine hybrids. The best primer set, Toll_R, targeting the putative Toll gene, showed little to no bias when used for DNA amplification from hybrid ticks. Thus, Toll gene can be further used for hybrid detection.

## 1. Introduction

After mosquitoes, ixodid ticks are considered one of the main vectors of arboviruses. The species composition of ticks in the local focus of arboviral infection can affect not only its epizootic potential but also the local risk of infection. Due to different ecology, features of the life cycle, and periods of activity, different species of ixodid ticks have different vector competence. Climate change (global warming), environmental factors, and anthropogenic impacts (landscape transformation and reduction in livestock numbers) are often listed as probable causes of changes in tick distribution [1].

The main vectors and reservoirs of tick-borne encephalitis virus (TBEV) are *Ixodes ricinus* (Europe, European part of Russia) and *Ixodes persulcatus* (northeastern Europe, Russia and Asia) ticks, whose ranges correspond to the TBEV area. The western part of the habitat of *I. persulcatus* overlaps with that of *I. ricinus*, forming a large sympatric area of these two species in the East European Plain and Baltic countries. In recent years, considerable changes in the distribution and abundance of these ticks in northern Europe and in the northern European part of Russia have been reported, which has led to changes in the ecoepidemiology of TBE and other tick-borne diseases in some places [2,3].

For the first time in 1998, it was shown that these tick species have no reproductive barrier, and as a result, crossing between them with the appearance of hybrids is possible [4]. According to data, reciprocal crossing of males and females is associated with a decrease in the percentage of fertile eggs, while the hybrid generation is sterile. This reproductive isolation in the absence of the morphophysiological barrier can considerably limit the abundance of *I. ricinus* and *I. persulcatus* within the sympatry area [2].

The effects of hybridization between arthropod vectors of disease remain largely unexplored. Interspecies genetic exchange may affect the biology of the vectors, the interaction with hosts, and the pathogens transmitted. For example, in mosquitoes from the *Culex pipiens* complex, it has been shown that hybridization of two bioforms of *C. pipiens* with *C. quinquefasciatus* significantly affects West Nile virus infection, dissemination, and particularly, transmission of the virus in hybrids compared to parental species [5]. Thus, hybridization of ticks can affect not only the spread of ticks but also the properties of natural foci of arbovirus infections; therefore, the study of hybrids of the main TBEV vectors seems highly relevant.

Earlier, we obtained hybrids of ticks generated by crossing *I. persulcatus* females and *I. ricinus* males under laboratory conditions, and then we studied the morphology [6,7]. Since species identification of larvae and nymphs is based on quantitative features, we were able to measure species-specific parameters of larvae and nymphs of *I. ricinus*, *I. persulcatus*, and their hybrids and calculate discriminant functions. The obtained discriminant equations with very high classification effectiveness helped us to prove the existence of hybrids in nature in the sympatry area of *I. persulcatus* and *I. ricinus* ticks in Karelia. The species identification of adult *I. persulcatus* and *I. ricinus* ticks is mostly based on the in vivo determination of the species by qualitative features. In this case, we are unable to use any statistical methods of differentiation [7], and only a laborious subjective assessment remains, which depends largely on the experience of a specialist. For quick and objective differentiation of hybrid ticks in natural populations, genetic markers are necessary.

The most common genetic markers used for species determination is mitochondrial (mtRNA) and ribosomal RNA (rRNA). Sequences of the internal transcribed spacer 2 (ITS2) are used particularly often. Eukaryotic cells contain hundreds of copies of ribosomal DNA in the chromosomes, with the ITS2 region located between 5.8S and 28S rRNAs [8]. Previously, different oligonucleotides targeting this region were used to identify species, study the genetic heterogeneity of ticks, and determine hybrids within specimens collected from natural habitats [2,9]. At the same time, several other markers have been used to study different aspects of ticks’ genetics. In order to uncover differences between *I. scapularis* populations, twelve nuclear markers were used across two studies along with mtRNA and rRNA [10,11].

There were several studies on genetic differentiation of the hybrid ticks. In the vast majority of such studies, approaches focused on sequencing of the rRNA [12,13,14,15]. Only in one case, the amplified fragment length polymorphisms method was used [16]. The first genetic markers that were used for *I. ricinus*, *I. persulcatus*, and their hybrid differentiation in natural populations were the mitochondrial gene of the first subunit of cytochrome c oxidase and ribosomal internal transcribed spacer 2 (ITS2) [12,17]. However, these markers were tested only on ticks from the sympatry zone in Estonia and never on pure hybrids obtained under laboratory conditions.

In the current study, we used laboratory-reared adult male and female hybrid ticks received from reciprocal crossings of *I. ricinus* and *I. persulcatus* ticks to assess the ability of different systems to differentiate hybrids and “pure” species.

## 2. Materials and Methods

### 2.1. Ticks

For the testing of primer sets, laboratory-reared first-generation adult hybrid ticks from reciprocal crossing of *I. persulcatus* and *I. ricinus* from laboratory colonies were used. It should be noted that laboratory colonies were originally maintained from females collected from territories of absolute dominance of one tick species: *I. persulcatus*—Republics of Karelia and Tuva, *I. ricinus*—Kaluga region. The process of hybrid rearing was described earlier [6]. Briefly, virgin *I. persulcatus* and *I. ricinus* females from laboratory colonies were mated with males of a different species and then were put onto a rabbit for feeding. Engorged females were kept in glass tubes with a humidity gradient [18], where they laid eggs and hybrid larvae hatched. Questing larvae and subsequent nymphs fed on laboratory mice. Engorged immature ticks were collected in glass tubes for molting into the next developmental stage. Hybrid females and males were separated into different tubes at the engorged nymph stage (according to the size) to avoid mating.

For initial oligonucleotide testing, field-collected *I. ricinus* and *I. persulcatus* ticks were used. Questing ticks were collected in 2018 by flagging in the four regions of Russia (Table 1): Republic of Karelia, Republic of Tuva (*I. persulcatus*), Kaliningrad region, and Voronezh region (*I. ricinus*) [19]. Ticks were identified using taxonomic keys [20], with identification being confirmed by ITS2 region sequencing using FITSMR-3SA primer set (Figure S1).

### 2.2. DNA Isolation

DNA was isolated using a diaGene kit for DNA extraction from cell culture (dia-m, Moscow, Russia) using a modified protocol. Ticks were homogenized in 300 μL of LB buffer (dia-m, Russia) using Tissue Lyser 2 (QIAGEN, Hilden, Germany) (12 min, frequency 25 s^−1^), after which the homogenate was centrifuged (5 min, 1500 rpm). Then, 3 μL of proteinase K (dia-m, Russia) and 6 μL of mercaptoethanol were added, and the mix was incubated at 56 °C overnight. Then, 420 μL of the sorption solution (dia-m, Russia) was added, and the mixture was transferred to a flow-through column and centrifuged (1 min, 15,700× *g*). The column was washed with 300 μL of WB1 buffer (dia-m, Russia) twice and then with 500 μL of Wash solution 2 (dia-m, Russia) twice with 1 min centrifugation (15,700× *g*) after each wash. The washed column was centrifuged one more time to remove residual buffers (1 min, 15,700× *g*) and placed into a new 1.5 mL tube. Thirty microliters of water was added, and after 3 min, DNA was eluted by centrifugation (1 min, 15,700× *g*).

### 2.3. PCR

PCR was performed directly from the extracted DNA using DreamTaq DNA polymerase (Thermo Fisher Scientific, Vilnius, Lithuania) according to the manufacturer’s instructions. A description of the primer sets used in the study is provided in Table 2 below.

### 2.4. PCR Product Purification, TA-Cloning, and Plasmid Purification and Sequencing

After PCR, each PCR product was gel-purified with a QIAGEN gel extraction kit (QIAGEN, Germany) according to the manufacturer’s instructions, and the downstream purity was confirmed through gel electrophoresis.

TA cloning was performed using an InsTAclone PCR Cloning kit (Thermo Scientific, Lithuania) according to the manufacturer’s instructions. Briefly, randomly selected white plaques (5–10 for each primer set tested) were transferred to 3 mL of LB medium overnight for growth. Plasmids with insertions were then purified from the bacterial culture using a GeneJet Plasmid Miniprep kit (Thermo Scientific, Lithuania) according to the manufacturer’s instructions.

Sequencing was carried out from either purified PCR products or from purified plasmid in both directions with BigDye Terminator v.3.1 Cycle Sequencing Kit (Thermo Fisher Scientific, Lithuania) on the ABI PRISM 3130 (Applied Biosystems, Waltham, MA, USA) genetic analyzer. The obtained sequences were viewed and assembled into *.fasta files using the SeqMan v. 7.0.0 software (DNAstar, Madison, WI, USA) and deposited into GenBank under the accession numbers: MZ725049-MZ725170, MZ725171-MZ725187, MZ713272-MZ713363.

### 2.5. qPCR

qPCR was carried out directly from isolated DNA as described in the work of Kovalev and co-authors [17] using reagents (reaction buffer, dNTP, MgCl_2_ and polymerase) from the qPCR reaction kit (Syntol, Moscow, Russia) on the Bio-Rad CFX96 Real-Time System (Bio-Rad, Hercules, CA, USA). Analysis of the amplification curves and species determination was performed according to the methods of Kovalev and co-authors [13,17].

### 2.6. Bioinformatics

Consensus sequences for Toll_R and lysozyme were constructed by aligning all obtained sequences for each species and performing the “extract consensus” procedure with the SeqMan v. 7.0.0 software (DNAstar, Madison, WI, USA). Pairwise distances were calculated with the MEGA 10.1.7 program [22]. For phylogenetic analysis, sequences were aligned in MEGA 10.1.7 using the ClustalW algorithm with default parameters. The obtained alignments were used to construct maximum likelihood phylogenetic trees in MEGA using 1000 bootstrap replicates [22].

Statistical analyses were carried out using Fischer’s exact test, using the expected distribution of the same size for a control group from classical Mendel’s inheritance scheme (1:0 or 1:1).

## 3. Results

### 3.1. Testing ITS Primer Sets

The most common marker currently used for phylogenetic studies of ticks is the ITS2 region. There are various primer sets targeting this region and a vast amount of the data present in the GenBank, which allows quick and easy species determination. Moreover, there are few systems targeting ITS regions specifically made for *Ixodes* hybrid differentiation. One of those systems was designed (and then improved) by Kovalev and co-authors to study specifically hybrids of *I. persulcatus* and *I. ricinus* ticks in natural populations. It used difference in the Ct values for specie-specific probe during qPCR to discriminate between pure species, hybrid ticks, and hybrids with different level of pure species introgression [13,17]. However, the system was only tested on the mixes with different quantities of the non-hybrid *I. ricinus* and *I. persulcatus* DNA and was never tested on laboratory-obtained hybrid ticks.

Here, we tested this system on laboratory-obtained hybrid ticks. Hybrid ticks were obtained in the laboratory by reciprocal crossing of *I. persulcatus* and *I. ricinus*. From each crossing, 2 male and 2 female hybrid ticks were used for testing. As a positive control for the system, 8 non-hybrid ticks of parental species were used. It should be noted that only F1 hybrids were used in our work.

The obtained results are summarized in Table 3. Non-hybrid ticks identification with the system agreed with both morphological and genetical identification done by us prior to the work. Out of eight hybrid ticks tested, only one of them (♀ h604) was identified correctly as F1 hybrid (Hybrid 1:1 according to Kovalev et al.’s classification). Five hybrid ticks (3 ♀ and 2 ♂) were identified as hybrids with different levels of pure species introgression (hybrid 1:2 to hybrid 1: ∞, according to Kovalev et al. classification). Two male F1 hybrid ticks were identified as *I. persulcatus*.

It is worth mentioning that this system worked especially poorly with male hybrid ticks. While in females, Ct delta stayed within 2, and one of the ticks was identified as F1 hybrid correctly, in male ticks, preferential amplification of the maternal DNA is notable. Male hybrids h606 and h730 were identified as their mothers’ species, while in male hybrids h423-1 and h423-2 delta Ct was about 10 in favor of *I. ricinus DNA* amplification.

In order to test if such a poor result in hybrid identification is a result of the oligonucleotide design itself or the bad choice of the target gene, we tested other well-known primer pairs targeting the ITS2 region (FITSMR-3SA and JB9A-3SA, Table 2). As there is no qPCR designed for these pairs, we used a molecular cloning approach to identify hybrids.

Individual tick DNA was amplified with selected ITS2 primers, and the obtained PCR product was TA-cloned. Six to ten colonies representing individual molecular clones were selected randomly for each primer pair. Each molecular clone was sequenced, and its species was identified by placement in the phylogenetic tree (Figures S2–S17). The obtained results are summarized in Table 4.

After hybridization, Mendel’s distribution (1:1) of the ITS2 region by species is expected, assuming the simplest inheritance scheme: ribosomal DNA (rDNA) is located in one cluster in one chromosome. However, the obtained data contradict this assumption. In most individual hybrids, there was a statistically significant difference (Fisher’s exact test) between the obtained distributions of molecular clones and Mendel’s distribution (1:1). Moreover, this occurred in both of the primer sets used in the study.

Additionally, in male ticks obtained from the ♀ *I. ricinus* × ♂ *I. persulcatus* crosses (h423-1 and h423-2), the *I. ricinus* ITS variant was predominantly observed, while in male ticks from the ♀ *I. persulcatus* × ♂ *I. ricinus* crosses (h606 and h730), only the *I. persulcatus* ITS variant was obtained. Thus, mostly maternal ITS2 variants were sequenced in male ticks; we found only one parental ITS2 clone out of 60 molecular clones in male hybrid ticks. It also agrees with data we observed using the qPCR system previously, where we detected preferable amplification of the maternal DNA.

It is known that the ITS2 region is located in rDNA repeats, which are present in hundreds of copies [8]. While there are no data on the distribution of rDNA on the chromosomes of *I. ricinus* and *I. persulcatus*, in *I. scapularis*, it is located on several chromosomes, including the X chromosome [23]. Localization of the rDNA on multiple chromosomes in *I. ricinus* and *I. persulcatus* can explain occasional extraction of the parental rDNA fragment. At the same time, data obtained in the work suggest that a major part of rDNA is X-chromosome linked. Indeed, although there are no data on the genetic sex determination of *I. ricinus* and *I. persulcatus*, in the studied *Ixodes* tick species, males are either a heterogametic sex (XY—sex chromosomes) or have just one X chromosome, while females always carry two X chromosomes [24]. In both of these cases, X-chromosome linked inheritance can explain the dominance of maternal rDNA in male hybrid ticks.

To test our X-linkage hypothesis, we analyzed the maternal:paternal distribution of molecular clones from males and females (Table 5). The X-chromosome linkage hypothesis predicts a 1:0 distribution of molecular clones in male ticks and a 1:1 ratio in female ticks. Statistical analysis using Fischer’s exact test shows that there is no statistically significant difference in the maternal:paternal distribution in males and females from the one expected from the rDNA X-linkage hypothesis. Thus, our data suggest that a vast amount of the ITS2 region (and a vast amount of rDNA copies, because rDNA is usually inherited as a whole) is located on the X chromosome.

Overall, such data make any oligonucleotides that target rDNA unacceptable for the determination of male *I. persulcatus*:*I. ricinus* hybrids, as there will be a high probability that such a test will result in false-negative results.

### 3.2. Searching for Prospective Genes for Hybrid Determination

The next goal was to find gene-specific oligonucleotides that will be convenient to use for hybrid determination. Our oligonucleotides of interest should satisfy three conditions: the target gene must not be located on the sex chromosome, oligonucleotides must have little to no bias in gene amplification of different species in the mixture, and the sequence of the amplified fragment must be divergent enough to allow reliable species determination by general bioinformatics methods.

Because our above data suggest that the ITS region may be associated with the sex chromosome, we decided to not use any rRNA amplicons in our work. Analysis of the available literature showed several oligonucleotides for non-ribosomal genes for *Ixodes scapularis* (Table 2). Additionally, we designed oligonucleotides for putative actin, odorant receptor, juvenile hormone methyltransferase (JH-MT), and Toll-like receptor genes based on the sequences found in the *I. scapularis* genome database (Table S1) [25]. To verify the ability of the primer sets to differentiate between species, we sequenced amplicons obtained from field-collected ticks. These ticks were collected in non-sympatric areas of Russia, and each tick species was determined both morphologically by taxonomic keys and genetically by sequencing the ITS2 region. In all cases, the results of determining the species morphologically matched the genetic determination results.

First, the ability of the aforementioned primer sets to amplify fragments from the *I. ricinus* and *I. persulcatus* ticks was tested and the ability to differentiate these species based on sequence divergence and overall amplicon length was estimated. Ticks No 420 (*I. ricinus*) and 338-2 (*I. persulcatus*) were used for this experiment (Table 1). We failed to amplify PCR fragments using the Serpin 2, Ixoderin, and OdorR primer sets, most likely because they were selected based on the *I. scapularis* DNA sequence. These primer pairs were not analyzed further. For amplified fragments, we analyzed the obtained sequences to estimate divergence between *I. ricinus* and *I. persulcatus* variants (Tables S2–S5). The actin gene was the most similar between this species (Table S2), and, combined with low amplicon length, it had little potential for hybrid differentiation and was not used in further research. Because Toll_full length is 1403 nucleotides, thus making it inconvenient to sequence it, we used the Toll_R amplicon, located within Toll_full length, for further research.

**Figure 1:**
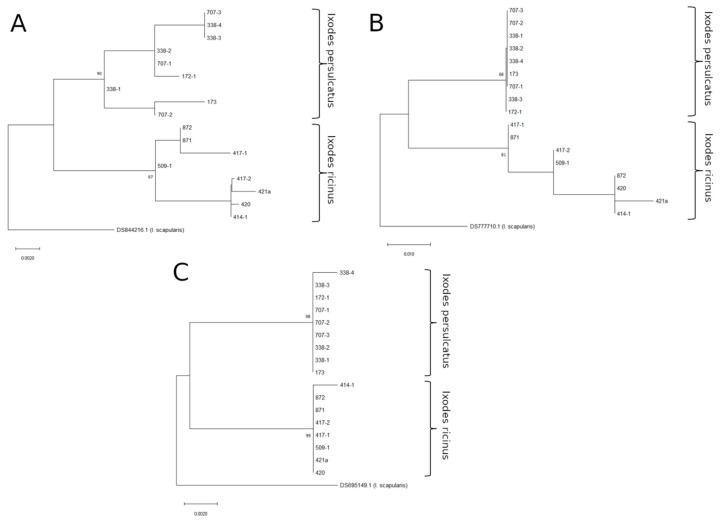
Maximum likelihood phylogenetic trees of the non-hybrid ticks, constructed using lysozyme (**A**), JH_MT (**B**), and Toll_R (**C**) amplicon sequences. The percentage of trees (>70%) in which the associated taxa clustered together (after performing 1000 bootstrap replicates) is shown next to the branches.

To more thoroughly inspect the ability of Toll_R, JH_MT, and lysozyme fragments to differentiate between tick species, we sequenced these genes for a number of *I. ricinus* and *I. persulcatus* ticks collected from various non-sympatric locations in Russia (Table 1). For all three primer sets, we were able to amplify DNA from both male and female *I. persulcatus* and *I. ricinus* ticks, suggesting that none of these target genes is located on the Y chromosome. To determine the ability of these markers to differentiate the species, maximum likelihood phylogenetic trees were constructed (Figure 1). In all of the trees constructed, the obtained sequences were divided into *I. ricinus* and *I. persulcatus* clades according to earlier data from the ITS2 gene fragment and identification with taxonomic keys. However, the confidence in the identification was not the same for all trees. The tree constructed using Toll_R gene fragment sequences had the best support for both *I. ricinus* (99%) and *I. persulcatus* (98%) clades. The tree based on the lysozyme gene fragment also had high support (90% and 97%), and the tree based on the JH_MT sequence had relatively low support for *I. ricinus/I. persulcatus* clades (68% and 81%). Based on these data, we chose Toll and lysozyme gene fragments as candidates for hybrid determination.

### 3.3. Testing Lysozyme and Toll_R Amplicons as a Hybrid Differentiator

In the final part of the work, we used oligonucleotide pairs to identify hybrids. In this experiment, we used an approach similar to the first approach with two modifications: we took fewer molecular clones per tick (4–5 for each amplicon) and tested only male hybrid ticks, as both of our concerns (X-chromosome location and amplification bias) can still be tested using such an approach. The obtained results (Figures S18–S25) are summarized in Table 6.

With non-hybrid DNA used as a matrix, a primer set targeting the lysozyme gene was able to successfully amplify both *I. persulcatus* and *I. ricinus* sequences. However, an *I. persulcatus:I. ricinus* molecular clone ratio of 1:18 suggests that this primer set clearly preferentially amplifies *I. ricinus* DNA in hybrid ticks. Nevertheless, the maternal to paternal ratio remained indistinguishably close to 1:1 in male hybrid ticks, suggesting a classical pattern of Mendelian inheritance for the lysozyme gene. Thus, the lysozyme gene is not located on the X chromosome and can be used for hybrid differentiation, although the primer set used in our work is unsuitable for hybrid differentiation because of preferential amplification of the *I. ricinus* gene variant.

In case of the Toll_R primer set, although there was a trend toward preferential amplification of *I. persulcatus* gene variant, the difference from classical Mendel’s distribution (1:1) was not significant. The maternal:paternal molecular clone distribution (12:8) shows no significant difference from a classical Mendelian distribution and was significantly different from the distribution expected for X chromosome-linked inheritance (1:0). These data suggest that Toll gene inheritance is not X chromosome-dependent and that the Toll_R primer set shows little to no bias when amplifying DNA from hybrid ticks. Thus, the Toll_R primer set can be used for hybrid differentiation using a molecular cloning approach without further modification.

## 4. Discussion

Various markers associated with mitochondrial DNA and rRNA have been shown to be useful in ixodid species discrimination [9,21]. These markers have well-described protocols, show very high rates of correct species determination [26], and have many sequences in GenBank to compare target sequences, so it was natural to use such markers not only for species determination but also for hybrid differentiation [12,13,14,15,17]. However, hybrid determination is complicated. Mitochondrial DNA markers can only be used for the identification of the maternal species, as mtDNA is transmitted through the maternal line in the vast majority of cases. While rRNA would be an ideal marker for hybrid determination in general, there is a study that shows that in *I. scapularis* ticks, rDNA is partially associated with sex chromosomes [23]. It was also suggested that the ITS2 region (which is part of rDNA [27]) may be located on the X chromosome in both *I. persulcatus* and *I. pavlovskiy* [15]. In hard ticks, sex is determined either by the XX-XY scheme or XX-XO scheme [24], and even if rDNA is only partly associated with an X chromosome, it may lead to false-negative mistakes in male hybrid identification. Because information on the chromosome distribution of the rDNA in other *Ixodes* tick species is unavailable, tests with laboratory-reared hybrids must be done to ensure that target regions for genetic hybrid determination are not located within the X chromosome.

The goal of the current study was to find a genetic marker that would allow us to reliably detect *I. ricinus* × *I. persulcatus* hybrid ticks. To achieve this goal, unlike in previous works, we used hybrid ticks derived from crossing laboratory-cultured *I. ricinus* and *I. persulcatus*. Moreover, in our work, we used reciprocal crossing, allowing us to differentiate between preferential gene amplification and sex-chromosome linked inheritance.

First, we tested well-known systems that target the ITS2 region within nuclear rRNA [9,17,21]. Our data show that all three tested systems are not suitable for *I. ricinus* × *I. persulcatus* hybrid determination. All systems showed especially high biases during amplification of the DNA from male hybrid ticks, with preference toward amplification of the maternal DNA. Our data suggest that a vast number of ITS2-coding copies localize on the X chromosome of the *I. ricinus* and *I. persulcatus* ticks. The ITS2 region is a part of the rDNA cistron that apart from ITS2 also encodes 18S, 5.8S, and 28S rRNA [27]. Thus, all these rRNAs are likely to be at least partly linked with the X chromosome.

More cytogenetic data on both *I. ricinus* and *I. persulcatus* are needed to uncover exact rDNA localization in *I. ricinus* and *I. persulcatus* ticks and accurately predict ITS2 inheritance mechanisms in hybrids. Nevertheless, in our opinion, data presented here undermine usability of rDNA as a tool for accurate differentiation of *I. ricinus* × *I. persulcatus* hybrid ticks. 

Previously, rDNA sequences were used to identify *I. ricinus* × *I. persulcatus* [12] and *I. persulcatus* × *I. pavlovskyi* [13,15] hybrid ticks in nature. In situations where rDNA gene regions may be located on sex chromosomes, the data must be interpreted with extreme caution. In several studies, ratios of the species rDNA in the hybrid tick were measured either by qPCR or by molecular cloning approaches. The authors assumed that the expected allele ratio in F1 hybrid ticks would be 1:1 [12]. Based on observed ratios, the authors concluded the existence of F2 hybrids and backcrosses in natural populations [12,13]. However, our data show that at least for *I. ricinus* × *I. persulcatus* hybrids, rDNA ratios obtained by either cloning or qPCR of the ITS2 region significantly differ from the 1:1 ratio. It shows that without a clear knowledge of species genetics, rDNA ratios cannot be interpreted reliably to detect F2 hybrids and backcrosses, and further research on this problem is needed using different approaches.

In the second part of the study, we tried to find novel candidate genes that were suitable for tick hybrid differentiation. Out of several oligonucleotide pairs tested, we were able to successfully amplify gene fragments from both *I. ricinus* and *I. persulcatus* ticks from four genes: putative lysozyme, actin, juvenile hormone methyltransferase, and Toll-like receptor. Fragments of the lysozyme and Toll genes were the best in species identification using maximum likelihood phylogenetic trees, making lysozyme and Toll_R primer pairs usable for further studies on pure *I. ricinus* and *I. persulcatus* tick populations. Note that the lysozyme primer pair was previously used to investigate variation within populations of *I. scapularis* in the USA [11].

In the final part of the work, we studied the ability of lysozyme and Toll gene sequences to differentiate tick hybrids. Our data suggest that neither of these genes are located on the X chromosome. However, the primer set targeting lysozyme was biased toward amplifying the *I. ricinus* gene variant in the hybrids, while the Toll_R primer set showed no statistically significant bias. Thus, the Toll_R primer set can be used without modifications to determine hybrids using a molecular cloning approach.

To unveil *I. ricinus* and *I. persulcatus* population dynamics in sympatric regions, a tool for hybrid determination is needed. There have been several studies on this problem using both morphological and molecular approaches [6,7,12]. The main advantage of the molecular biology approach is that multiple ticks can be analyzed at the same time. However, in contrast to well-studied species determination approaches [26], determining hybrids poses a unique set of challenges, especially if the genetics of the parental species is poorly studied. Our work highlights the importance of using laboratory-obtained hybrids for testing in such cases.

## 5. Conclusions

In this work, laboratory-bred hybrid *I. ricinus* × *I. persulcatus* ticks were used to assess the ability of several gene primer sets to identify hybrid ticks. Our data show that three different systems relying on amplification of the ITS2 regions were unable to reliably distinguish laboratory-bred hybrid ticks. We hypothesize that it can be explained by the localization of a high number of the ITS2 copies in the X chromosome. Thus, using these genes and whole rDNA cistrone for hybrid identification can lead to false results.

Both Toll receptor and lysozyme genes can be used as a differentiation tool for *I. ricinus* × *I. persulcatus* hybrid ticks. Of all tested oligonucleotide pairs tested in our work, Toll_R was the best and can be used for hybrid identification without further modification.

## Figures and Tables

**Table 1:** Non-hybrid ticks used in the work.

Tick Specimen №	Tick Species	Sex	Collection Place	GPS
172-1	*Ixodes persulcatus*	♀	Russia, Republic of Tuva	51.9923° 94.0842°
173	*Ixodes persulcatus*	♀		51.6283° 94.4292°
338-1	*Ixodes persulcatus*	♂	Russia, Republic of Karelia	62.0586° 33.9427°
338-2	*Ixodes persulcatus*	♂		
338-3	*Ixodes persulcatus*	♂		
338-4	*Ixodes persulcatus*	♂		
707-1	*Ixodes persulcatus*	♀	Russia, Republic of Karelia	62.0747° 33.9495°
707-2	*Ixodes persulcatus*	♀		
707-3	*Ixodes persulcatus*	♀		
414-1	*Ixodes ricinus*	♀	Russia, Voronezh region	51.7801° 39.4085°
420	*Ixodes ricinus*	♀		
509-1	*Ixodes ricinus*	♂		
421a	*Ixodes ricinus*	♂	Russia, Voronezh region	51.7850° 39.4054°
417-1	*Ixodes ricinus*	♂	Russia, Kaliningrad region	55.1839° 20.8572°
417-2	*Ixodes ricinus*	♂		
871	*Ixodes ricinus*	♀	Russia, Kaliningrad region	55.1585° 20.8437°
872	*Ixodes ricinus*	♀		

**Table 2:** Primer sets used in this work.

Primer Set Name	Oligonucleotide	Oligonucleotide Sequence	Primer Set Temperature	Reference	Amplicon Length
FITSMR-3SA	FITSMR	5′-ccagtattcatcggggacgc-3′	55 °C	[9,21]	≈660
	3SA	5′-ctaagcggtggatcactcgg-3′			
JB9A-3SA	JB9A	5′-gcactatcaagcaacacgactc-3′	52 °C	[9]	≈1100
	3SA	5′-ctaagcggtggatcactcgg-3′			
Lysozyme	Lys_eSNP1F	5′-tgtctttggcttggatcgtc-3′	55 °C	[11]	512
	Lys_eSNP1R	5′-attcttccacctgccctacg-3′			
Serpin 2	Serp2Ae1-6_F	5′-ttacgctcccgacgttattc-3′	50–55 °C *	[11]	651
	Serp2Ae1-6_R	5′-ttcgagggatcaaacaggtc-3′			
Ixoderin	IxodBe2-3F	5′-acacgtatgcctcaaagtgg-3′	50–55 °C *	[11]	502
	IxodBe2-3R	5′-gcactatatccagcgggaag-3′			
Actin	Act_E6_F	5′-ggagcacccgctccaggta-3′	55 °C	- **	268
	Act_E6_R	5′-ctgtaattgcagctgccggac-3′			
OdorR	OdorR_F	5′-attccccacatcgcgcaa-3′	50–55 °C *	-	536
	OdoR_R	5′-ccctcatttctcagattctagcg-3′			
JH_Met	JH_MT_F	5′-gtctctaaacagatggtcgact-3′	52 °C	-	308
	JH_MT_R	5′-agactcacctcggcgta-3′			
Toll_full	Toll_F	5′-ctcgaacgtcatgaagact-3′	50 °C	-	1403
	Toll_end_R	5′-acttttgcggctatctgtttat-3′			
Toll_L	Toll_F	5′-ctcgaacgtcatgaagact-3′	50 °C	-	811
	Toll_mid_R	5′-ttcgatccagttgttacccaggct-3′			
Toll_R	Toll_mid_F	5′-gcgattgaatctctggaggg-3′	50 °C	-	739
	Toll_end_R	5′-acttttgcggctatctgtttat-3′			

* failed to amplify *I. ricinus* and *I. persulcatus* with temperatures in the range. ** designed in the current work.

**Table 3:** Results of the hybrid differentiation using method previously described by Kovalev et al. [17].

Tick Specimen No	Sex	Tick Species	Ct *I. persulcatus*	Ct *I. ricinus*	Ct Delta	Tick Species (as Determined by Kovalev et al.’s System)
h604	♀	hybrid ♀ *I.persulcatus* × ♂ *I.ricinus*	15.93	15.49	0.44	Hybrid 1:1
h605	♀	hybrid ♀ *I.persulcatus* × ♂ *I.ricinus*	17.24	18.27	1.03	Hybrid 1:2
h606	♂	hybrid ♀ *I.persulcatus* × ♂ *I.ricinus*	23.00	nd *	- **	*I. persulcatus*
h730	♂	hybrid ♀ *I.persulcatus* × ♂ *I.ricinus*	15.05	nd	-	*I. persulcatus*
h422-1	♀	hybrid ♀ *I.ricinus* × ♂ *I.persulcatus*	20.73	22.51	1.78	Hybrid 1:4
h422-2	♀	hybrid ♀ *I.ricinus* × ♂ *I.persulcatus*	17.79	18.99	1.20	Hybrid 1:2
h423-1	♂	hybrid ♀ *I.ricinus* × ♂ *I.persulcatus*	29.25	19.17	10.08	Hybrid 1:∞
h423-2	♂	hybrid ♀ *I.ricinus* × ♂ *I.persulcatus*	30.62	20.10	10.52	Hybrid 1:∞
707-2	♀	*I. persulcatus*	14.33	nd	-	*I. persulcatus*
707-3	♀	*I. persulcatus*	15.05	nd	-	*I. persulcatus*
338-1	♂	*I. persulcatus*	15.09	nd	-	*I. persulcatus*
173	♀	*I. persulcatus*	14.95	nd	-	*I. persulcatus*
872	♀	*I. ricinus*	nd	15.20	-	*I. ricinus*
417-2	♂	*I. ricinus*	nd	16.88	-	*I. ricinus*
421a	♂	*I. ricinus*	nd	17.01	-	*I. ricinus*
414-1	♀	*I. ricinus*	nd	14.60	-	*I. ricinus*

* amplification is not detected. ** Ct delta cannot be calculated due to one of the channels reporting no amplification.

**Table 4:** Ratios of *I. persulcatus*:*I. ricinus* molecular clones of the ITS2 region obtained for hybrid ticks.

Crossing		♀ *I. persulcatus* × ♂ *I. ricinus*				♀ *I. ricinus* × ♂ *I. persulcatus*			
**Individual tick**		h604 ♀	h605 ♀	h606 ♂	h730 ♂	h422-1 ♀	h422-2 ♀	h423-1 ♂	h423-2 ♂
**Clones from FITSMR-3SA ratio**	Individual	6:0 *	4:2	6:0 *	10:0 *	10:0 *	7:1	0:9 *	1:8
	Total by sex	10:2		16:0 *		17:1 *		1:17 *	
**Clones from JB9A-3SA ratio**	Individual	8:2	5:5	10:0 *	6:0 *	6:0 *	5:0	0:6 *	0:5
	Total by sex	13:7		16:0 *		11:0 *		0:11 *	

* statistically significant difference (*p* < 0.05) compared to distribution expected from classical Mendel’s inheritance scheme (1:1).

**Table 5:** Ratios of molecular clones of the ITS2 region obtained for hybrid ticks.

Crossing	Both ♀ *I. persulcatus* × ♂ *I. ricinus* and ♀ *I. ricinus* × ♂ *I. persulcatus*	
	**FITSMR-3SA ITS in**	
	♀	♂
***I. persulcatus*:*I. ricinus* ratio**	27:3 *	17:17
**maternal:paternal ratio**	11:19 †	33:1 #
	**JB9A-3SA ITS in**	
	♀	♂
***I. persulcatus*:*I. ricinus* ratio**	24:7 *	16:11
**maternal:paternal ratio**	13:18 †	27:0 #
	**Total ITS in**	
	♀	♂
**maternal:paternal ratio**	24:37 †	60:1 #

*—statistically significant difference (*p* < 0.05) compared to distribution expected from classical Mendel’s inheritance scheme (1:1). †—no statistically significant difference (*p* > 0.05) compared to distribution expected from X-chromosome linkage scheme (1:1). #—no statistically significant difference (*p* > 0.05) compared to distribution expected from X-chromosome linkage scheme (1:0).

**Table 6:** Ratios of *I. persulcatus*:*I. ricinus* molecular clones of the lysozyme and Toll region obtained for hybrid ticks.

Crossing		♀ *I. persulcatus* × ♂ *I. ricinus*		♀ *I. ricinus* × ♂ *I. persulcatus*	
**Individual tick**		h606 ♂	h730 ♂	h423-1 ♂	h423-2 ♂
**Clones from Lysozyme ratio**	Individual	1:4	0:5	0:5	0:4
	Total	1:18 *			
**Lysozyme total** **maternal:paternal ratio**		10:9 ^‡^			
**Clones from Toll_R ratio**	Individual	3:2	4:1	2:3	3:2
	Total	12:8			
**Toll_R total** **maternal:paternal ratio**		12:8 ^‡^			

*—statistically significant difference (*p* < 0.05) compared to distribution expected from classical Mendel’s inheritance scheme (1:1). ^‡^—statistically significant difference (*p* < 0.05) compared to distribution (maternal:paternal) if inheritance is X chromosome dependent (1:0 for ♂).

## Data Availability

Obtained sequencing data were deposited in the GenBank database (MZ725049-MZ725170, MZ725171-MZ725187, MZ713272-MZ713363).

## Supplementary Materials

The following supporting information can be downloaded at: https://www.mdpi.com/article/10.3390/microorganisms10071306/s1, Figure S1: Phylogenetic tree of the ticks used for amplicon testing constructed by obtained ITS2 region sequences; Table S1: *Ixodes scapularis* gene fragments that were used in the nucleotide alignments and estimates of divergence; Table S2: Estimates of divergence between Actin gene fragment sequences; Table S3: Estimates of divergence between Toll gene fragment sequences (after amplification with Toll_Full primer set); Table S4: Estimates of divergence between juvenile hormone metiltranspherase gene fragment sequences; Table S5: Estimates of divergence between lysozyme gene fragment sequences; Figure S2: Maximum likelihood tree for the ITS gene fragments sequenced from molecular clones obtained from hybrid tick h604 after amplification with FITSMR-3SA primer set; Figure S3: Maximum likelihood tree for the ITS gene fragments sequenced from molecular clones obtained from hybrid tick h605 after amplification with FITSMR-3SA primer set; Figure S4: Maximum likelihood tree for the ITS gene fragments sequenced from molecular clones obtained from hybrid tick h606 after amplification with FITSMR-3SA primer set; Figure S5: Maximum likelihood tree for the ITS gene fragments sequenced from molecular clones obtained from hybrid tick h730 after amplification with FITSMR-3SA primer set; Figure S6: Maximum likelihood tree for the ITS gene fragments sequenced from molecular clones obtained from hybrid tick h422-1 after amplification with FITSMR-3SA primer set; Figure S7: Maximum likelihood tree for the ITS gene fragments sequenced from molecular clones obtained from hybrid tick h422-2 after amplification with FITSMR-3SA primer set; Figure S8: Maximum likelihood tree for the ITS gene fragments sequenced from molecular clones obtained from hybrid tick h423-1 ITS gene fragments with FITSMR-3SA primer set. Figure S9: Maximum likelihood tree for the ITS gene fragments sequenced from molecular clones obtained from hybrid tick h423-2 after amplification with FITSMR-3SA primer set; Figure S10: Maximum likelihood tree for the ITS gene fragments sequenced from molecular clones obtained from hybrid tick h604 after amplification with JB9A-3SA primer set; Figure S11: Maximum likelihood tree for the ITS gene fragments sequenced from molecular clones obtained from hybrid tick h605 after amplification with JB9A-3SA primer set; Figure S12: Maximum likelihood tree for the ITS gene fragments sequenced from molecular clones obtained from hybrid tick h606 after amplification with JB9A-3SA primer set; Figure S13: Maximum likelihood tree for the ITS gene fragments sequenced from molecular clones obtained from hybrid tick h730 after amplification with JB9A-3SA primer set; Figure S14: Maximum likelihood tree for the ITS gene fragments sequenced from molecular clones obtained from hybrid tick h422-1 after amplification with JB9A-3SA primer set; Figure S15: Maximum likelihood tree for the ITS gene fragments sequenced from molecular clones obtained from hybrid tick h422-2 after amplification with JB9A-3SA primer set; Figure S16: Maximum likelihood tree for the ITS gene fragments sequenced from molecular clones obtained from hybrid tick h423-1 after amplification with JB9A-3SA primer set; Figure S17: Maximum likelihood tree for the ITS gene fragments sequenced from molecular clones obtained from hybrid tick h423-2 after amplification with JB9A-3SA primer set; Figure S18: Maximum likelihood tree for the lysozyme gene fragments sequenced from molecular clones obtained from hybrid tick h730; Figure S19: Maximum likelihood tree for the lysozyme gene fragments sequenced from molecular clones obtained from hybrid tick h606; Figure S20: Maximum likelihood tree for the lysozyme gene fragments sequenced from molecular clones obtained from hybrid tick h423-1; Figure S21: Maximum likelihood tree for the lysozyme gene fragments sequenced from molecular clones obtained from hybrid tick h423-2; Figure S22: Maximum likelihood tree for the Toll_R gene fragments sequenced from molecular clones obtained from hybrid tick h730; Figure S23: Maximum likelihood tree for the Toll_R gene fragments sequenced from molecular clones obtained from hybrid tick h606; Figure S24: Maximum likelihood tree for the Toll_R gene fragments sequenced from molecular clones obtained from hybrid tick h423-1; Figure S25: Maximum likelihood tree for the Toll_R gene fragments sequenced from molecular clones obtained from hybrid tick h423-2.
