# Supplementary material for: Differentiation of Laboratory-Obtained Ixodes ricinus × Ixodes persulcatus Hybrid Ticks: Selection of Suitable Genes
Source: Microorganisms. 2022 Jun 27;10(7):1306. doi: 10.3390/microorganisms10071306 (PMC9323786; doi:10.3390/microorganisms10071306)

Figure S1. Phylogenetic tree of the ticks used for amplicon testing constructed by obtained ITS2 region sequences.

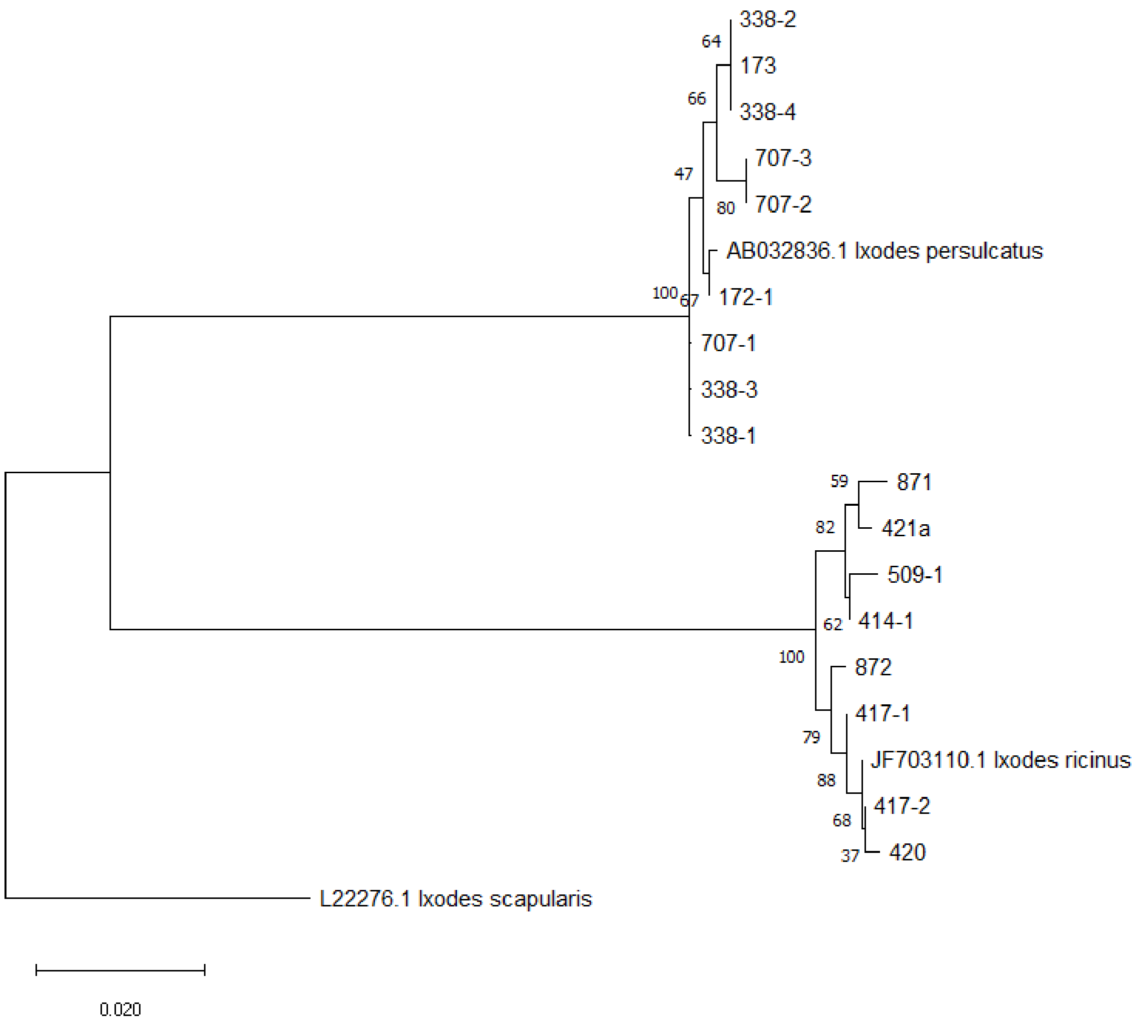

Table S1. *Ixodes scapularis* gene fragments that were used in the nucleotide alignments and estimates of divergence.

Gene	NCBI Acession number	Locus Tag
Lysozyme	DS844216.1	IscW_ISCW020680
Toll	DS695149.1	IscW_ISCW004495
JH_MT	DS777710.1	IscW_ISCW007168
Actin	DS643033.1	IscW_ISCW000900

Table S2. Estimates of Divergence between Actin gene fragment sqeuences. The number of base substitutions per site from between sequences are shown. Analyses were conducted using the Maximum Composite Likelihood model [22]. All ambiguous positions were removed for each sequence pair (pairwise deletion option). Evolutionary analyses were conducted in MEGA X [22]. Distance between *I. ricinus* and *I. persulacatus* are in green.

	№ 420 (*I. ricinus*)	338-2 (*I. persulcatus*)
DS643033.1 (*I. scapularis*)	0.00894	0.01335
№ 420 (*Ixodes ricinus*)		0.00894

Table S3. Estimates of Divergence between Toll gene fragment sequences (after amplification with Toll_Full primer set). The number of base substitutions per site from between sequences are shown. Analyses were conducted using the Maximum Composite Likelihood model [22]. All ambiguous positions were removed for each sequence pair (pairwise deletion option). Evolutionary analyses were conducted in MEGA X [22]. Distance between *I. ricinus* and *I. persulacatus* are in green.

	№ 420 (*I. ricinus*)	338-2 (*I. persulcatus*)
DS695149.1 (*I. scapularis*)	0.0227	0.0275
№ 420 (*I. ricinus*)		0.0211

Table S4. Estimates of Divergence between Juvenile hormone metiltranspherase gene fragment sequences. The number of base substitutions per site from between sequences are shown. Analyses were conducted using the Maximum Composite Likelihood model [22]. All ambiguous positions were removed for each sequence pair (pairwise deletion option). Evolutionary analyses were conducted in MEGA X [22]. Distance between *I. ricinus* and *I. persulacatus* are in green.

	№ 420 (*I. ricinus*)	338-2 (*Ixodes persulcatus*)
DS777710.1 (*I. scapularis*)	0.0686	0.0502
№ 420 (*I. ricinus*)		0.0598

Table S5. Estimates of Divergence between Lysozyme gene fragment sequences. The number of base substitutions per site from between sequences are shown. Analyses were conducted using the Maximum Composite Likelihood model [22]. All ambiguous positions were removed for each sequence pair (pairwise deletion option). Evolutionary analyses were conducted in MEGA X [22]. Distance between *I. ricinus* and *I. persulacatus* are in green.

	№ 420 (*I. ricinus*)	338-2 (*I. persulcatus*)
DS844216.1 *(I. scapularis)*	0.0283	0.0174
№ 420 (*I. ricinus*)		0.0200

Figure S2. Maximum likelihood tree for the ITS gene fragments sequenced from molecular clones obtained from hybrid tick **h604♀** after amplification with **FITSMR – 3SA** primer set. Sequences from hybrid tick are marked with a red dot. The tree with the highest log likelihood (-1444.38) is shown after performing 1000 bootstrap replicates. The percentage of trees (> 70%) in which the associated taxa clustered together is shown next to the branches. The tree is drawn to scale, with branch lengths measured in the number of substitutions per site. Evolutionary analyses were conducted in MEGA X [22]

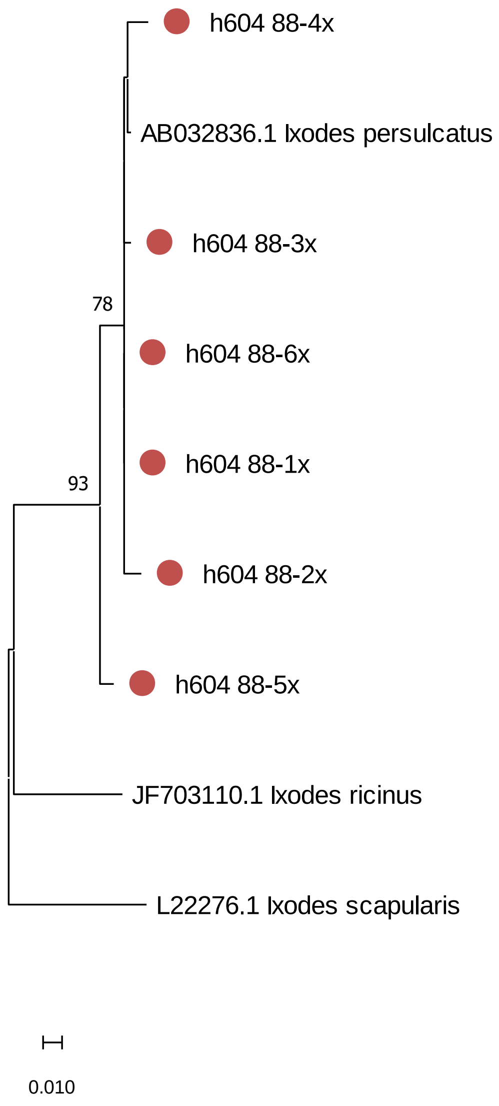

Figure S3. Maximum likelihood tree for the ITS gene fragments sequenced from molecular clones obtained from hybrid tick **h605♀** after amplification with **FITSMR – 3SA** primer set. Sequences from hybrid tick are marked with a red dot. The tree with the highest log likelihood (-1381.08) is shown after performing 1000 bootstrap replicates. The percentage of trees (> 70%) in which the associated taxa clustered together is shown next to the branches. The tree is drawn to scale, with branch lengths measured in the number of substitutions per site. Evolutionary analyses were conducted in MEGA X [22]

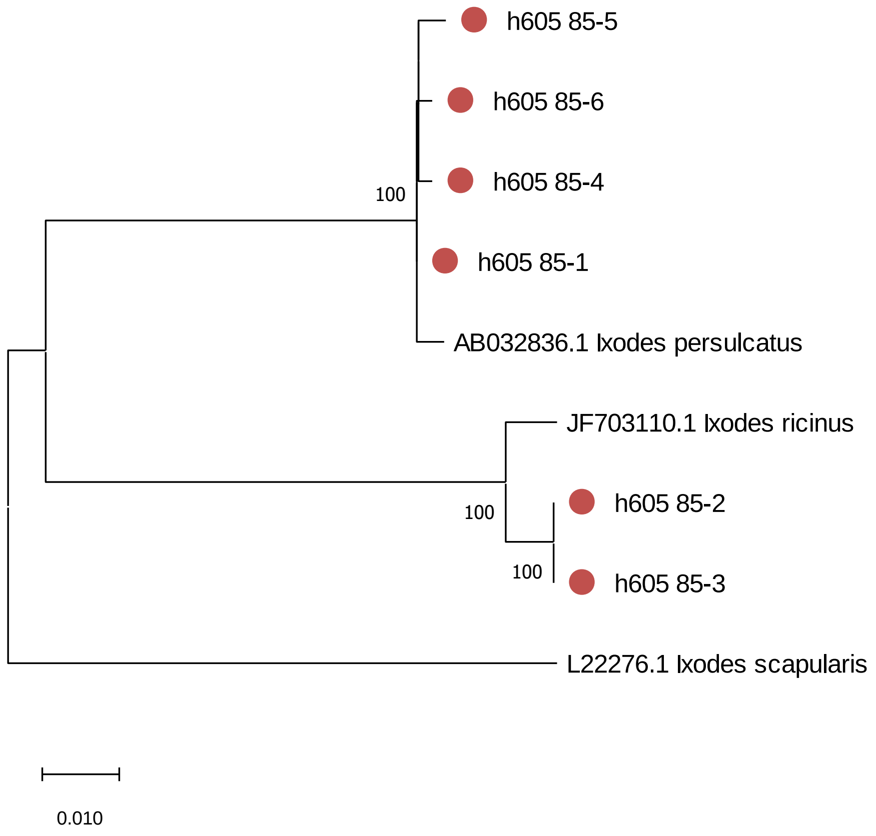

Figure S4. Maximum likelihood tree for the ITS gene fragments sequenced from molecular clones obtained from hybrid tick **h606♂** after amplification with **FITSMR – 3SA** primer set. Sequences from hybrid tick are marked with a red dot. The tree with the highest log likelihood (-1356.83) is shown after performing 1000 bootstrap replicates. The percentage of trees (> 70%) in which the associated taxa clustered together is shown next to the branches. The tree is drawn to scale, with branch lengths measured in the number of substitutions per site. Evolutionary analyses were conducted in MEGA X [22]

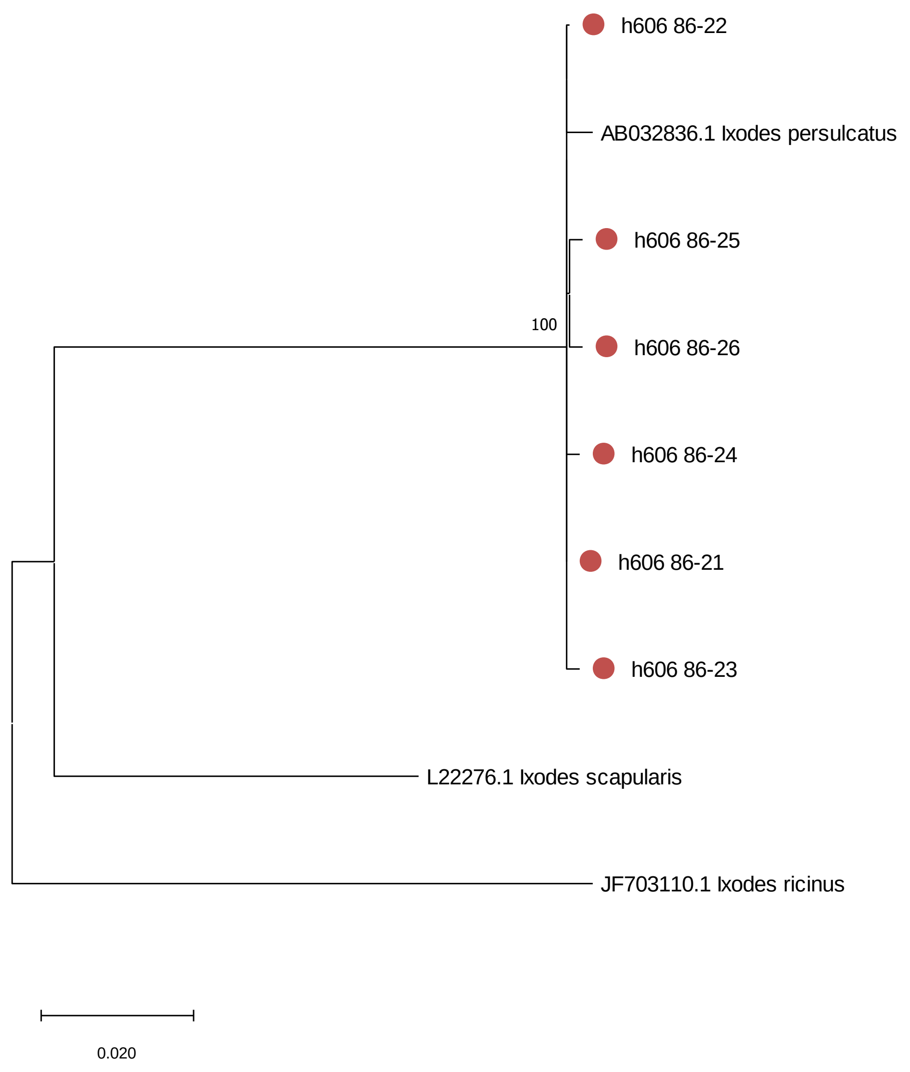

Figure S5. Maximum likelihood tree for the ITS gene fragments sequenced from molecular clones obtained from hybrid tick **h730♂** after amplification with **FITSMR – 3SA** primer set. Sequences from hybrid tick are marked with a red dot. The tree with the highest log likelihood (-1397.48) is shown after performing 1000 bootstrap replicates. The percentage of trees (> 70%) in which the associated taxa clustered together is shown next to the branches. The tree is drawn to scale, with branch lengths measured in the number of substitutions per site. Evolutionary analyses were conducted in MEGA X [22]

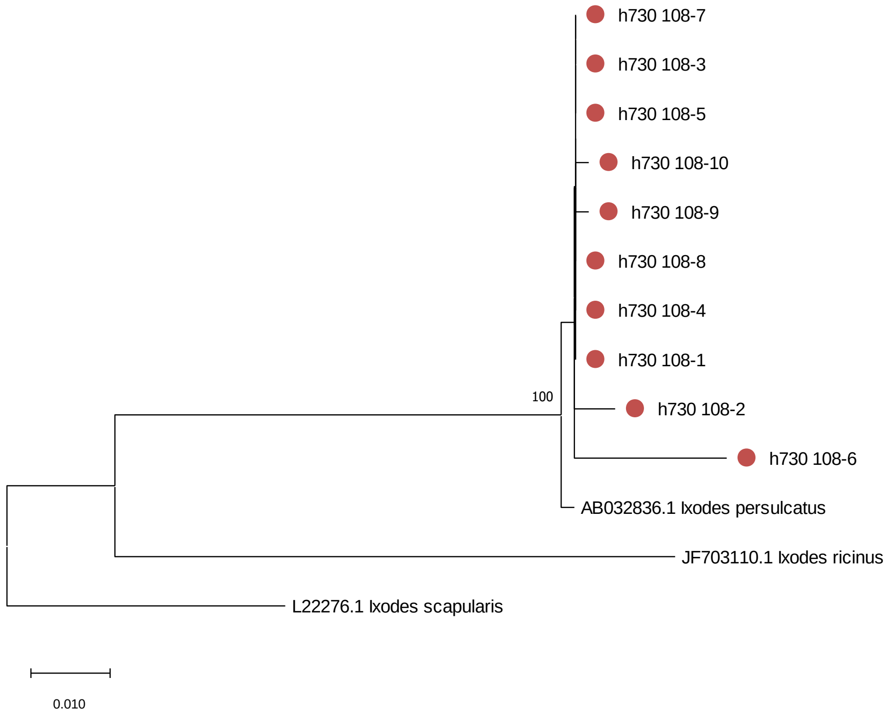

Figure S6. Maximum likelihood tree for the ITS gene fragments sequenced from molecular clones obtained from hybrid tick **h422-1♀** after amplification with **FITSMR – 3SA** primer set. Sequences from hybrid tick are marked with a red dot. The tree with the highest log likelihood (-1473.63) is shown after performing 1000 bootstrap replicates. The percentage of trees (> 70%) in which the associated taxa clustered together is shown next to the branches. The tree is drawn to scale, with branch lengths measured in the number of substitutions per site. Evolutionary analyses were conducted in MEGA X [22]

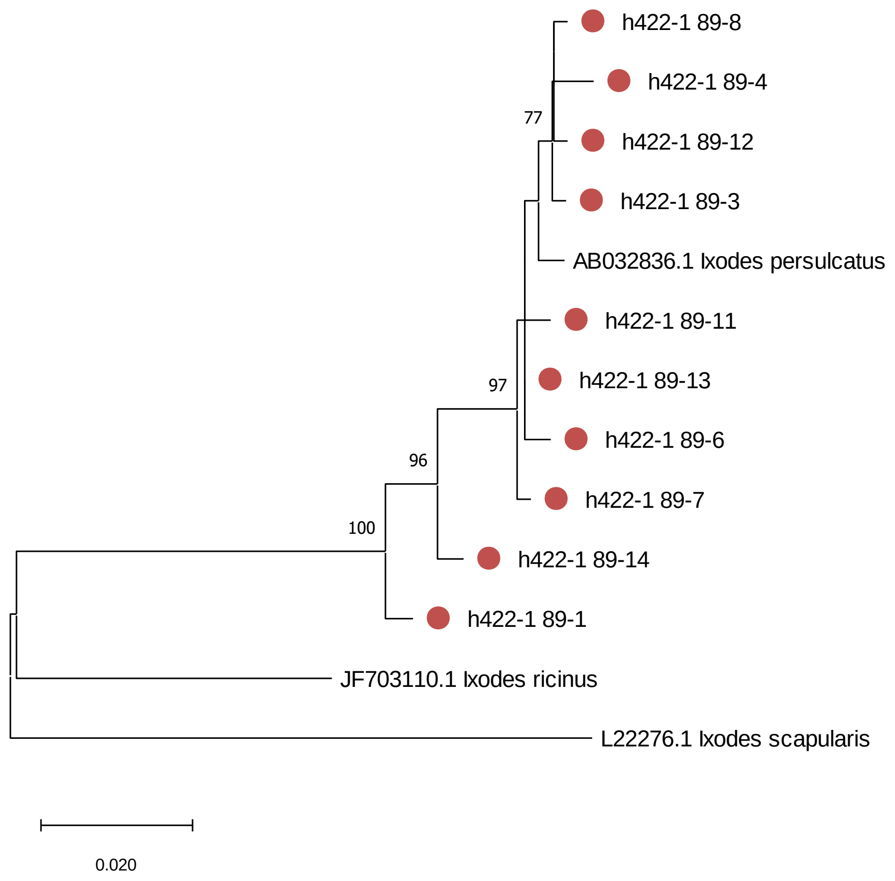

Figure S7. Maximum likelihood tree for the ITS gene fragments sequenced from molecular clones obtained from hybrid tick **h422-2♀** after amplification with **FITSMR – 3SA** primer set. Sequences from hybrid tick are marked with a red dot. The tree with the highest log likelihood (-1548.93) is shown after performing 1000 bootstrap replicates. The percentage of trees (> 70%) in which the associated taxa clustered together is shown next to the branches. The tree is drawn to scale, with branch lengths measured in the number of substitutions per site. Evolutionary analyses were conducted in MEGA X [22]

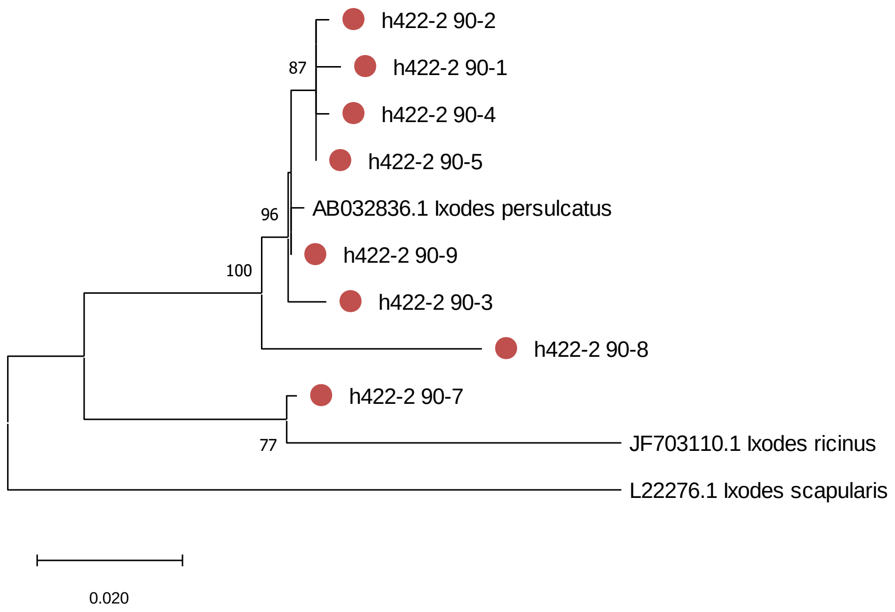

Figure S8. Maximum likelihood tree for the ITS gene fragments sequenced from molecular clones obtained from hybrid tick **h423-1♂** after amplification with **FITSMR – 3SA** primer set. Sequences from hybrid tick are marked with a red dot. The tree with the highest log likelihood (-1354.17) is shown after performing 1000 bootstrap replicates. The percentage of trees (> 70%) in which the associated taxa clustered together is shown next to the branches. The tree is drawn to scale, with branch lengths measured in the number of substitutions per site. Evolutionary analyses were conducted in MEGA X [22]

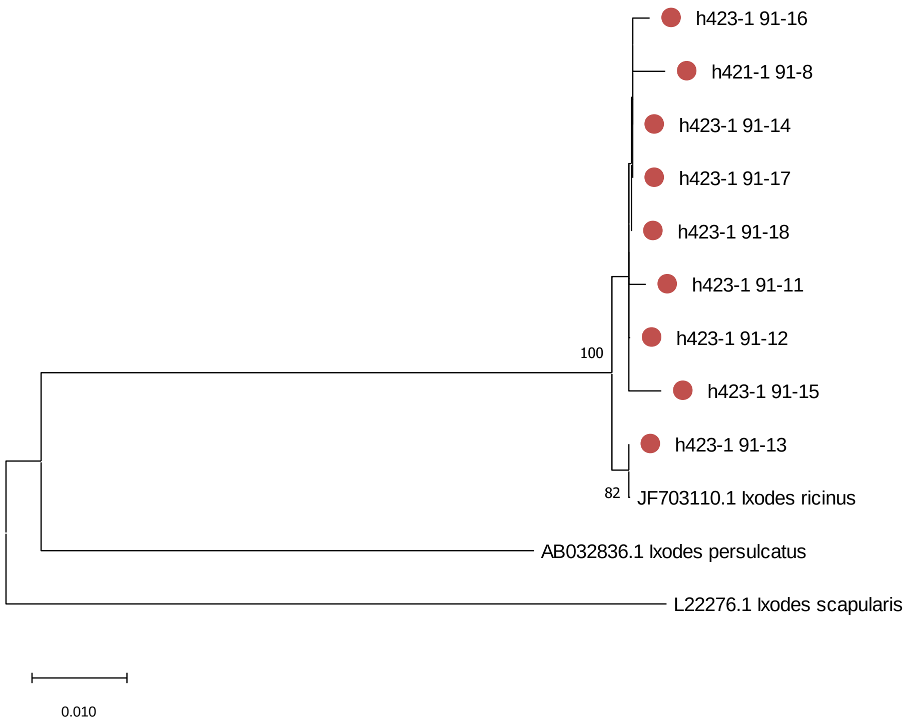

Figure S9. Maximum likelihood tree for the ITS gene fragments sequenced from molecular clones obtained from hybrid tick **h423-2**♂ after amplification with **FITSMR – 3SA** primer set. Sequences from hybrid tick are marked with a red dot. The tree with the highest log likelihood (-1516.34) is shown after performing 1000 bootstrap replicates. The percentage of trees (> 70%) in which the associated taxa clustered together is shown next to the branches. The tree is drawn to scale, with branch lengths measured in the number of substitutions per site. Evolutionary analyses were conducted in MEGA X [22]

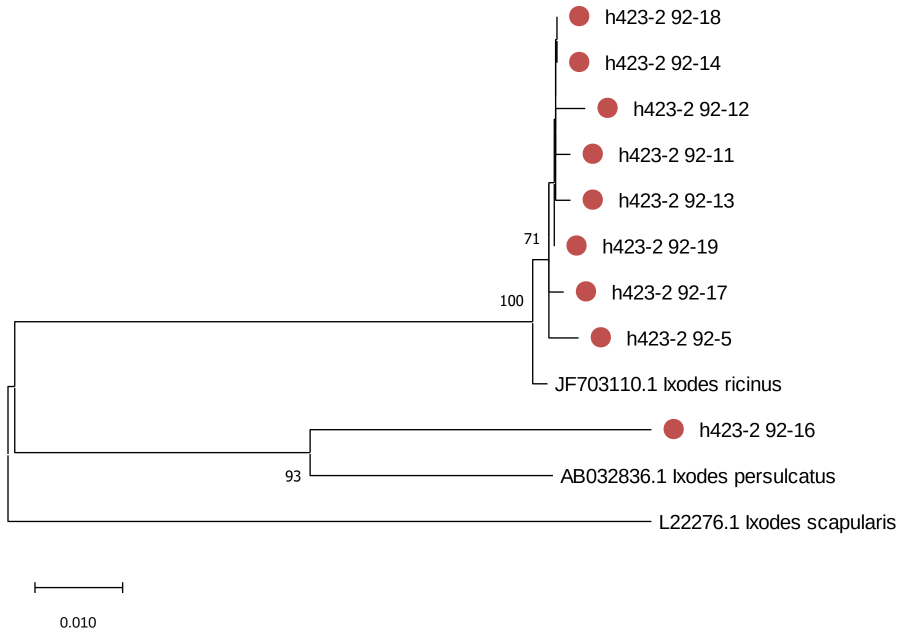

Figure S10. Maximum likelihood tree for the ITS gene fragments sequenced from molecular clones obtained from hybrid tick **h604♀** after amplification with **JB9A– 3SA** primer set. Sequences from hybrid tick are marked with a red dot. The tree with the highest log likelihood (-1500.98) is shown after performing 1000 bootstrap replicates. The percentage of trees (> 70%) in which the associated taxa clustered together is shown next to the branches. The tree is drawn to scale, with branch lengths measured in the number of substitutions per site. Evolutionary analyses were conducted in MEGA X [22]

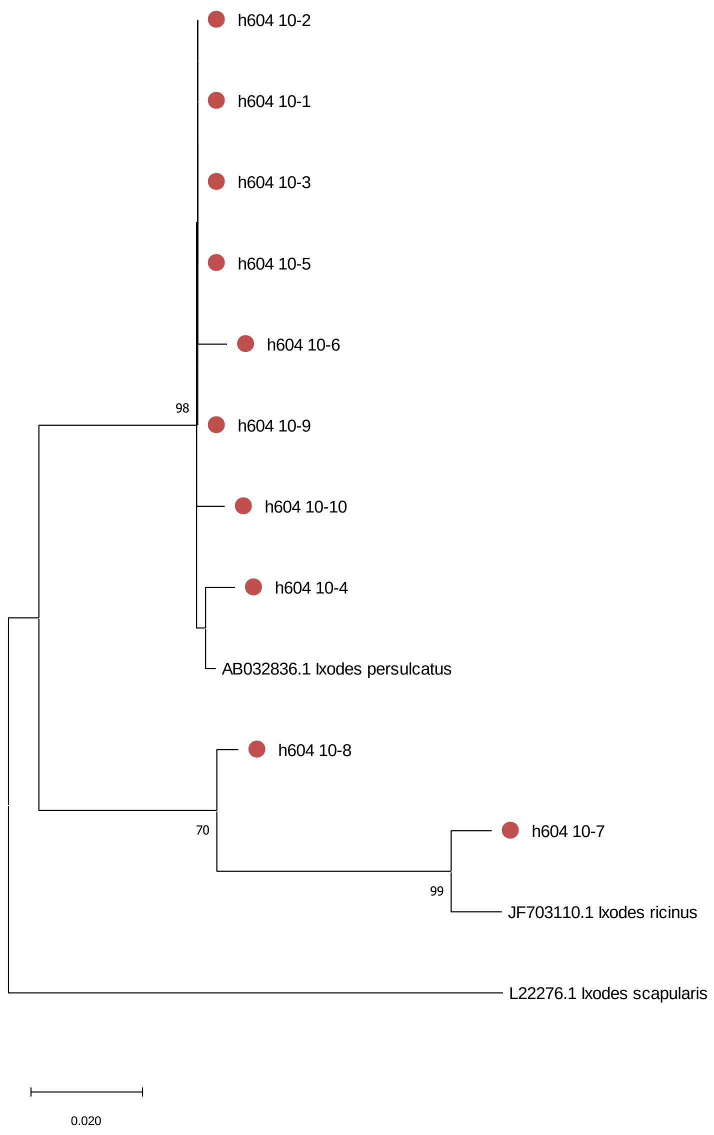

Figure S11. Maximum likelihood tree for the ITS gene fragments sequenced from molecular clones obtained from hybrid tick **h605♀** after amplification with **JB9A– 3SA** primer set. Sequences from hybrid tick are marked with a red dot. The tree with the highest log likelihood (-1450.09) is shown after performing 1000 bootstrap replicates. The percentage of trees (> 70%) in which the associated taxa clustered together is shown next to the branches. The tree is drawn to scale, with branch lengths measured in the number of substitutions per site. Evolutionary analyses were conducted in MEGA X [22]

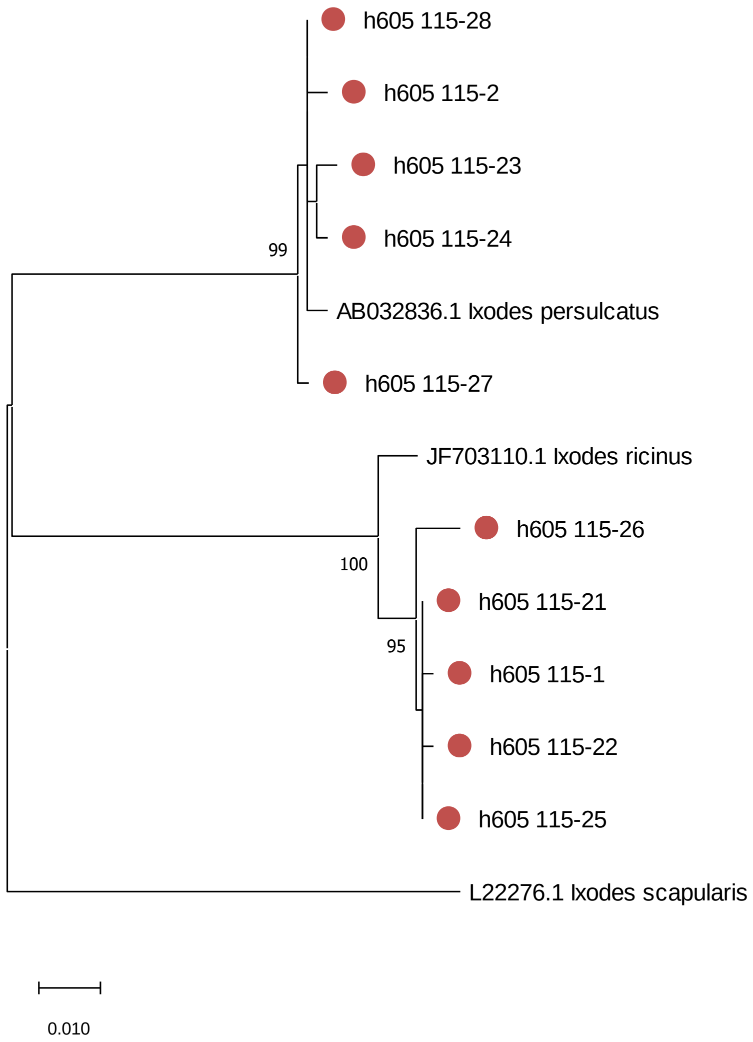

Figure S12. Maximum likelihood tree for the ITS gene fragments sequenced from molecular clones obtained from hybrid tick **h606♂** after amplification with **JB9A– 3SA** primer set. Sequences from hybrid tick are marked with a red dot. The tree with the highest log likelihood (-1379.25) is shown after performing 1000 bootstrap replicates. The percentage of trees (> 70%) in which the associated taxa clustered together is shown next to the branches. The tree is drawn to scale, with branch lengths measured in the number of substitutions per site. Evolutionary analyses were conducted in MEGA X [22]

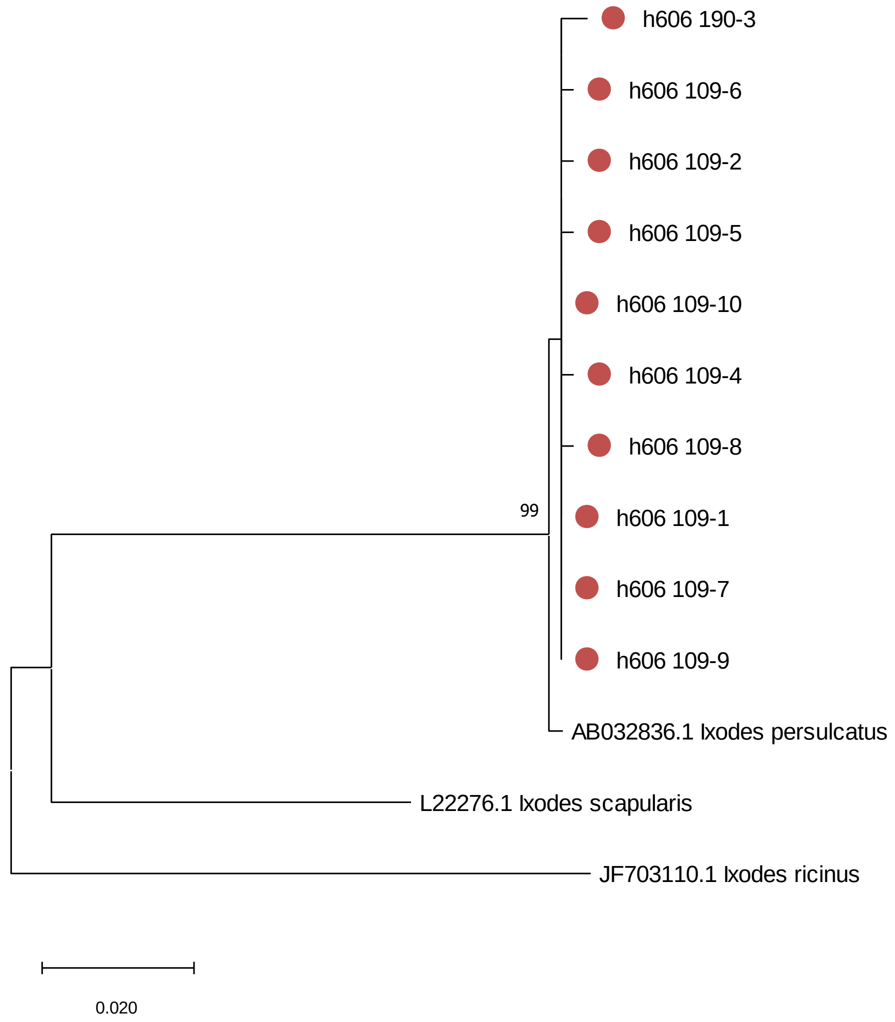

Figure S13. Maximum likelihood tree for the ITS gene fragments sequenced from molecular clones obtained from hybrid tick **h730♂** after amplification with **JB9A– 3SA** primer set. Sequences from hybrid tick are marked with a red dot. The tree with the highest log likelihood (-1349.89) is shown after performing 1000 bootstrap replicates. The percentage of trees (> 70%) in which the associated taxa clustered together is shown next to the branches. The tree is drawn to scale, with branch lengths measured in the number of substitutions per site. Evolutionary analyses were conducted in MEGA X [22]

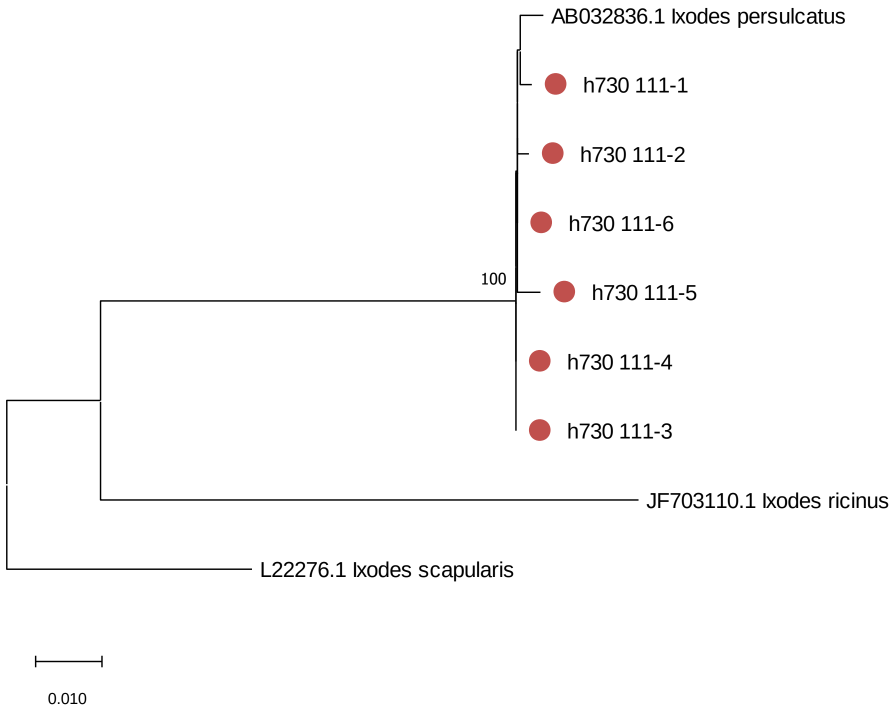

Figure S14. Maximum likelihood tree for the ITS gene fragments sequenced from molecular clones obtained from hybrid tick **h422-1**♀ after amplification with **JB9A– 3SA** primer set. Sequences from hybrid tick are marked with a red dot. The tree with the highest log likelihood (-1361.81) is shown after performing 1000 bootstrap replicates. The percentage of trees (> 70%) in which the associated taxa clustered together is shown next to the branches. The tree is drawn to scale, with branch lengths measured in the number of substitutions per site. Evolutionary analyses were conducted in MEGA X [22]

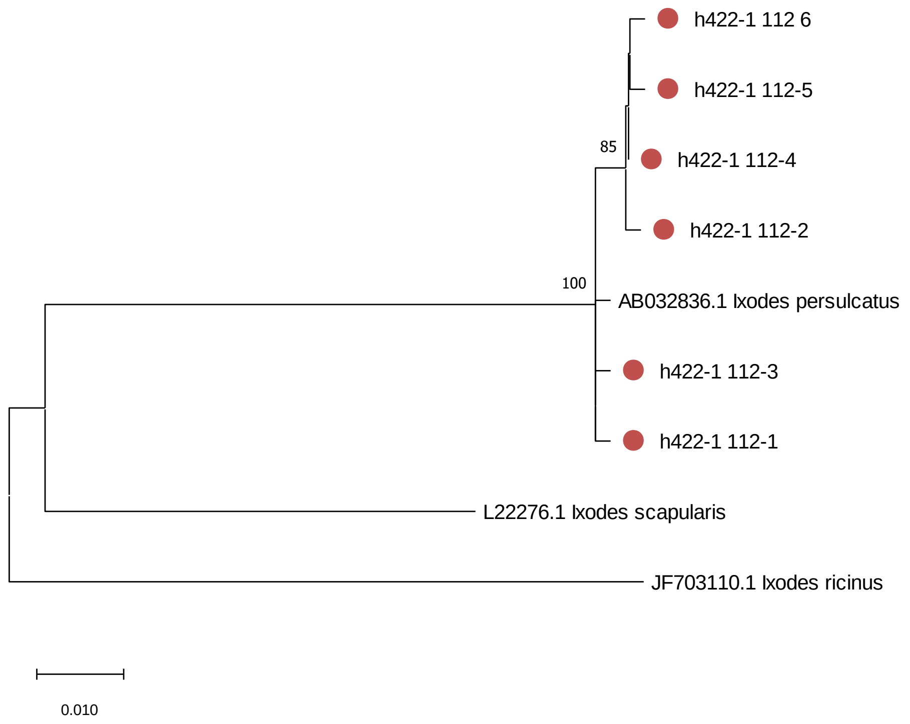

Figure S15. Maximum likelihood tree for the ITS gene fragments sequenced from molecular clones obtained from hybrid tick **h422-2♀** after amplification with **JB9A– 3SA** primer set. Sequences from hybrid tick are marked with a red dot. The tree with the highest log likelihood (-1351.12) is shown after performing 1000 bootstrap replicates. The percentage of trees (> 70%) in which the associated taxa clustered together is shown next to the branches. The tree is drawn to scale, with branch lengths measured in the number of substitutions per site. Evolutionary analyses were conducted in MEGA X [22]

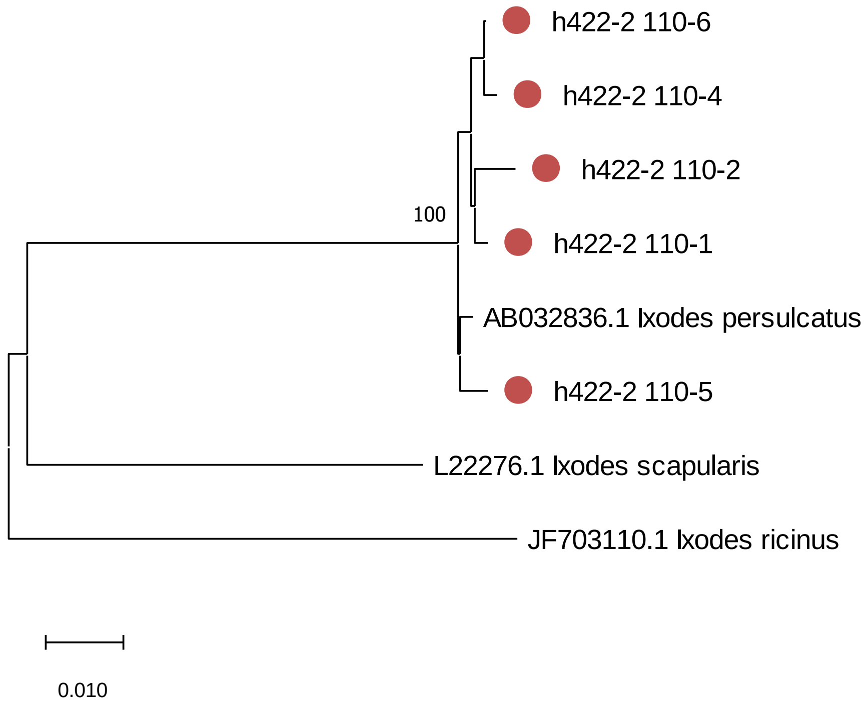

Figure S16. Maximum likelihood tree for the ITS gene fragments sequenced from molecular clones obtained from hybrid tick **h423-1♂** after amplification with **JB9A– 3SA** primer set. Sequences from hybrid tick are marked with a red dot. The tree with the highest log likelihood (-1349.87) is shown after performing 1000 bootstrap replicates. The percentage of trees (> 70%) in which the associated taxa clustered together is shown next to the branches. The tree is drawn to scale, with branch lengths measured in the number of substitutions per site. Evolutionary analyses were conducted in MEGA X [22]

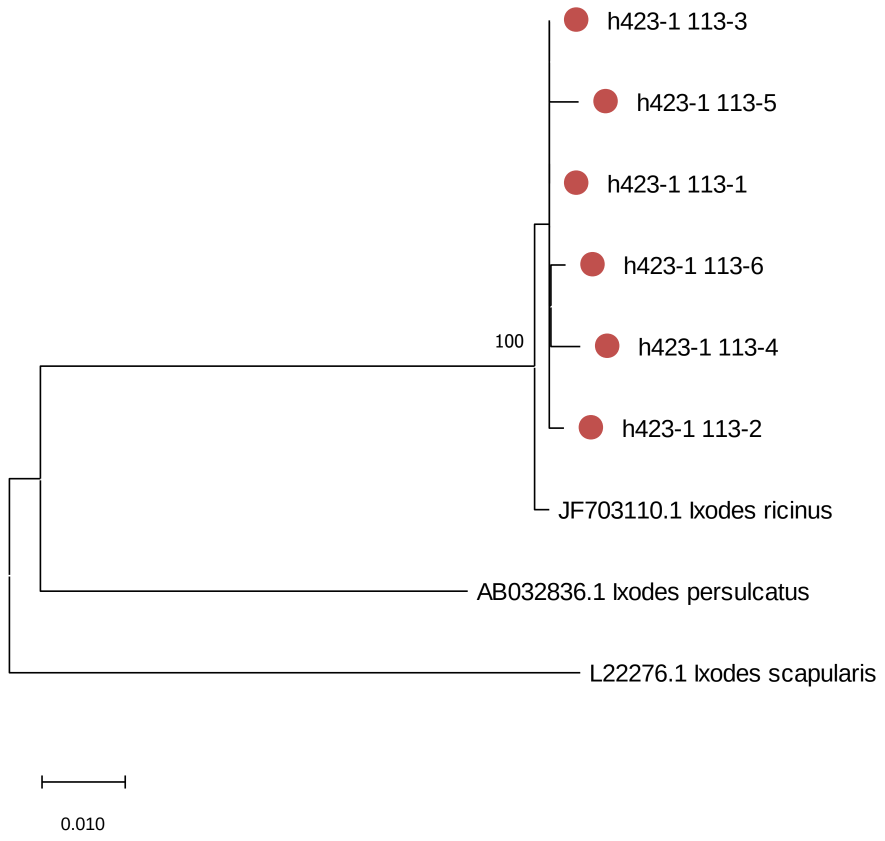

Figure S17. Maximum likelihood tree for the ITS gene fragments sequenced from molecular clones obtained from hybrid tick **h423-2**♂ after amplification with **JB9A– 3SA** primer set. Sequences from hybrid tick are marked with a red dot. The tree with the highest log likelihood (-1320.47) is shown after performing 1000 bootstrap replicates. The percentage of trees (> 70%) in which the associated taxa clustered together is shown next to the branches. The tree is drawn to scale, with branch lengths measured in the number of substitutions per site. Evolutionary analyses were conducted in MEGA X [22]

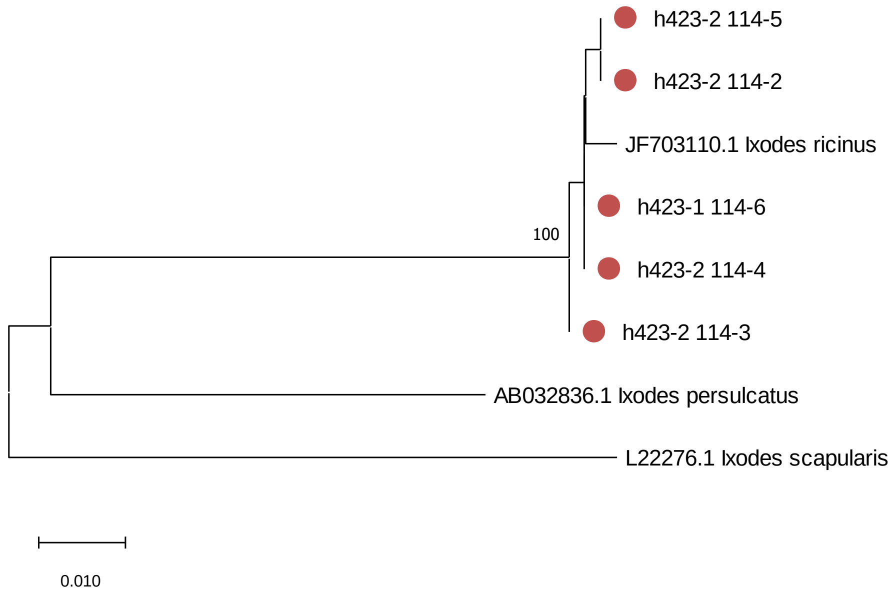

Figure S18. Maximum likelihood tree for the lysozyme gene fragments sequenced from molecular clones obtained from hybrid tick **h730♂**. Sequences from hybrid tick are marked with a red dot. The tree with the highest log likelihood (-901.55) is shown after performing 1000 bootstrap replicates. The percentage of trees (> 70%) in which the associated taxa clustered together is shown next to the branches. The tree is drawn to scale, with branch lengths measured in the number of substitutions per site. Evolutionary analyses were conducted in MEGA X [22]

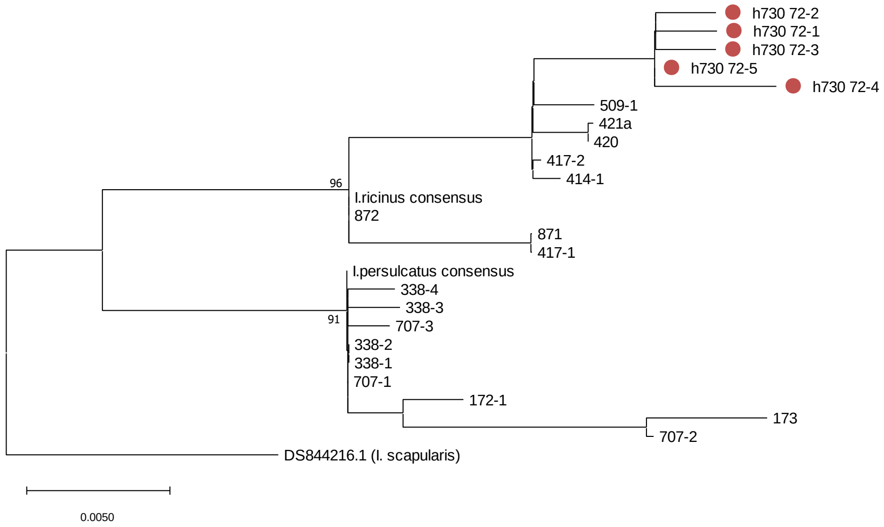

Figure S19. Maximum likelihood tree for the lysozyme gene fragments sequenced from molecular clones obtained from hybrid tick **h606♂**. Sequences from hybrid tick are marked with a red dot. The tree with the highest log likelihood (-908.46) is shown after performing 1000 bootstrap replicates. The percentage of trees (> 70%) in which the associated taxa clustered together is shown next to the branches. The tree is drawn to scale, with branch lengths measured in the number of substitutions per site. Evolutionary analyses were conducted in MEGA X [22]

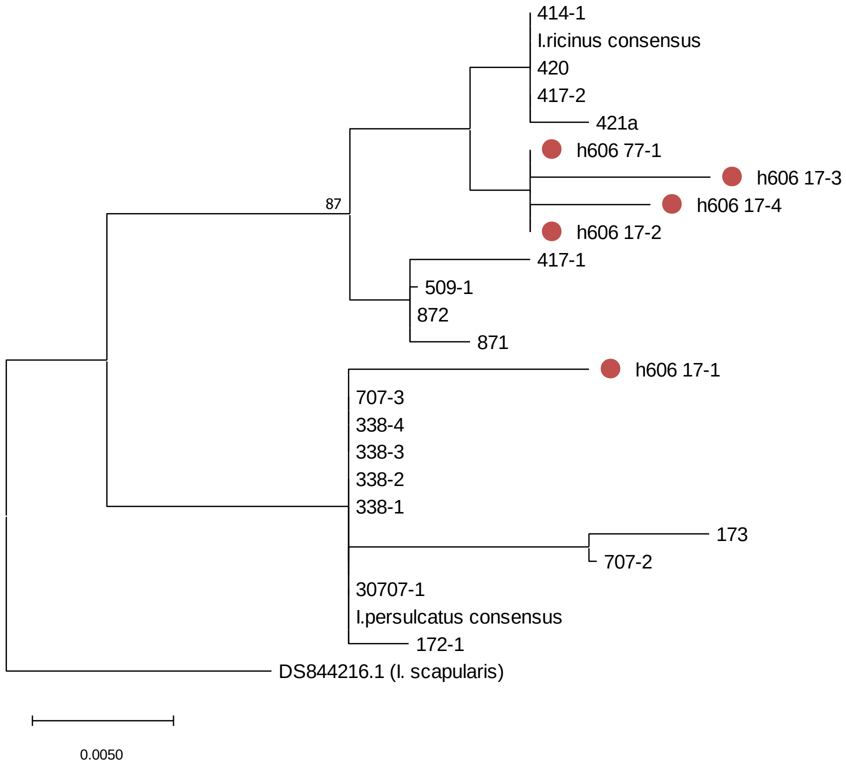

Figure S20. Maximum likelihood tree for the lysozyme gene fragments sequenced from molecular clones obtained from hybrid tick **h423-1**♂. Sequences from hybrid tick are marked with a red dot. The tree with the highest log likelihood (-935.66) is shown after performing 1000 bootstrap replicates. The percentage of trees (> 70%) in which the associated taxa clustered together is shown next to the branches. The tree is drawn to scale, with branch lengths measured in the number of substitutions per site. Evolutionary analyses were conducted in MEGA X [22]

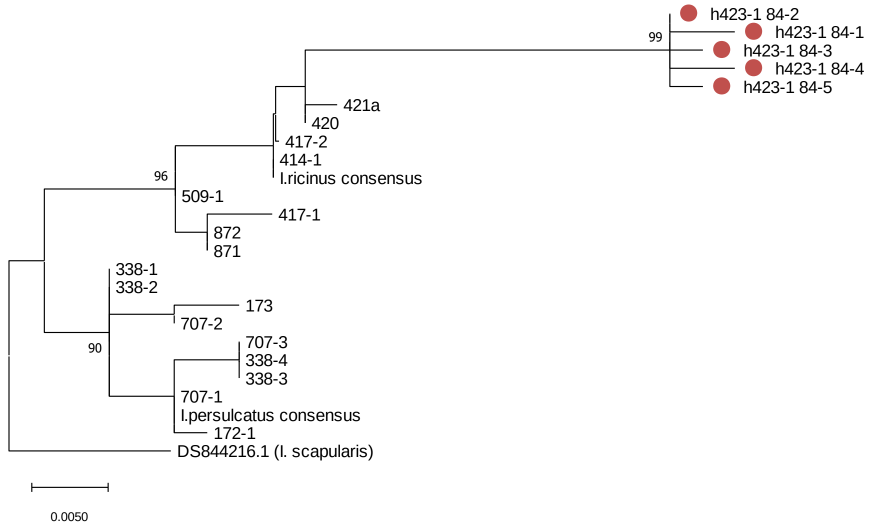

Figure S21. Maximum likelihood tree for the lysozyme gene fragments sequenced from molecular clones obtained from hybrid tick **h423-2**♂. Sequences from hybrid tick are marked with a red dot. The tree with the highest log likelihood (-941.10) is shown after performing 1000 bootstrap replicates. The percentage of trees (> 70%) in which the associated taxa clustered together is shown next to the branches. The tree is drawn to scale, with branch lengths measured in the number of substitutions per site. Evolutionary analyses were conducted in MEGA X [22]

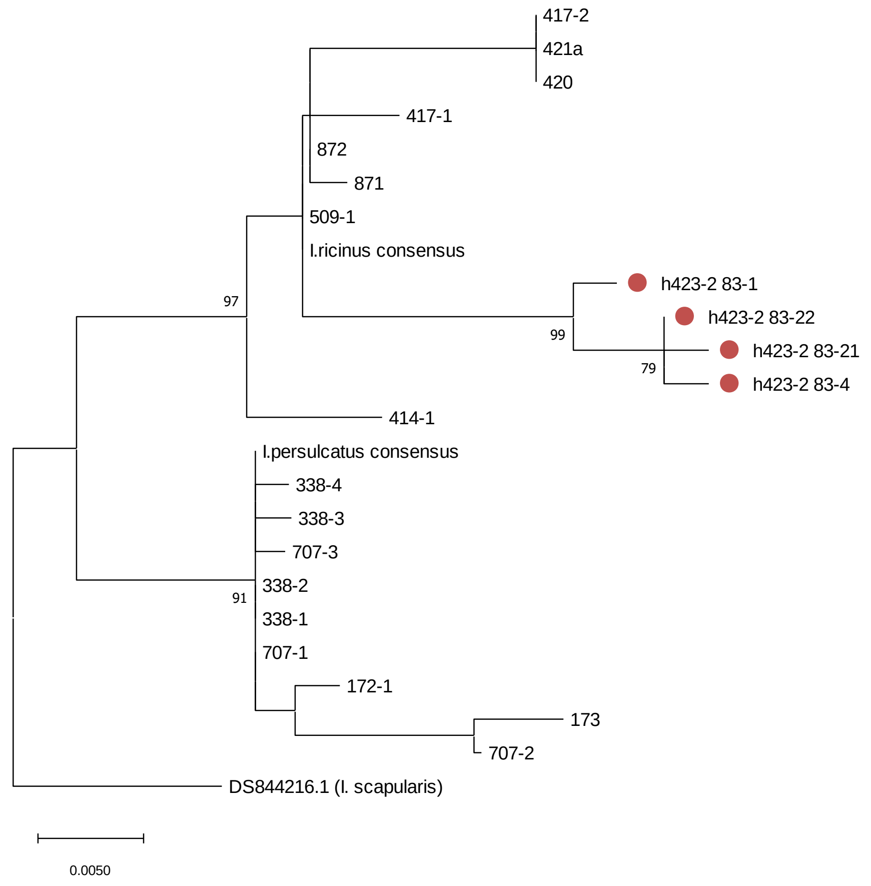

Figure S22. Maximum likelihood tree for the Toll_R gene fragments sequenced from molecular clones obtained from hybrid tick **h730**♂. Sequences from hybrid tick are marked with a red dot. The tree with the highest log likelihood (-1107.62) is shown after performing 1000 bootstrap replicates. The percentage of trees (> 70%) in which the associated taxa clustered together is shown next to the branches. The tree is drawn to scale, with branch lengths measured in the number of substitutions per site. Evolutionary analyses were conducted in MEGA X [22]

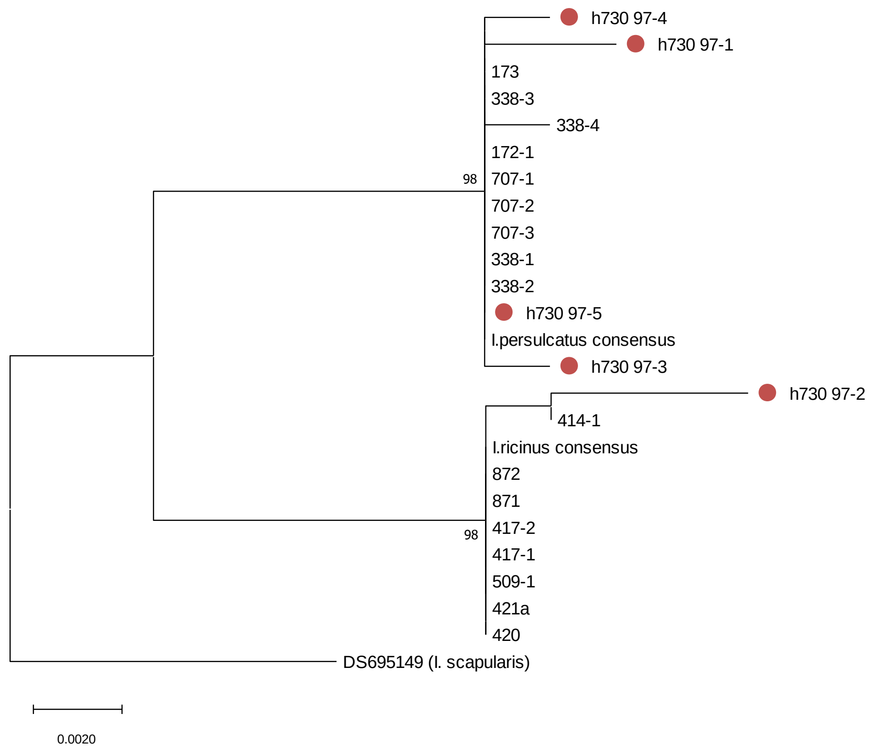

Figure S23. Maximum likelihood tree for the Toll_R gene fragments sequenced from molecular clones obtained from hybrid tick **h606**♂. Sequences from hybrid tick are marked with a red dot. The tree with the highest log likelihood (-1088.55) is shown after performing 1000 bootstrap replicates. The percentage of trees (> 70%) in which the associated taxa clustered together is shown next to the branches. The tree is drawn to scale, with branch lengths measured in the number of substitutions per site. Evolutionary analyses were conducted in MEGA X [22]

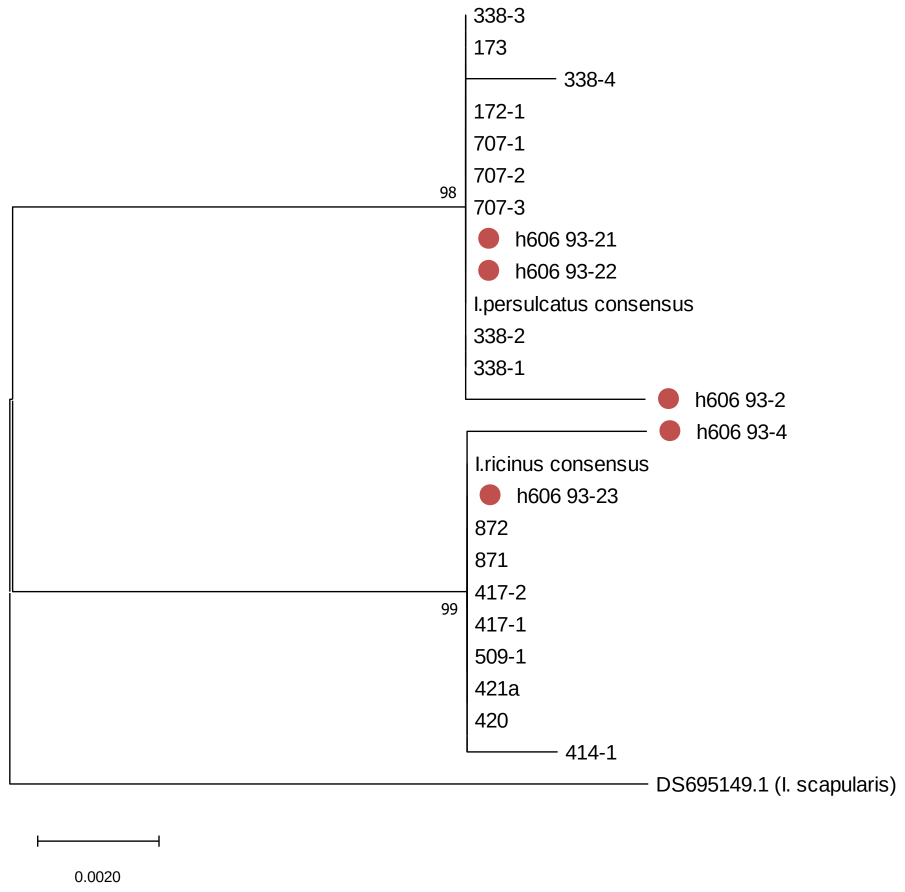

Figure S24. Maximum likelihood tree for the Toll_R gene fragments sequenced from molecular clones obtained from hybrid tick **h423-1**♂. Sequences from hybrid tick are marked with a red dot. The tree with the highest log likelihood (-1113.69) is shown after performing 1000 bootstrap replicates. The percentage of trees (> 70%) in which the associated taxa clustered together is shown next to the branches. The tree is drawn to scale, with branch lengths measured in the number of substitutions per site. Evolutionary analyses were conducted in MEGA X [22]

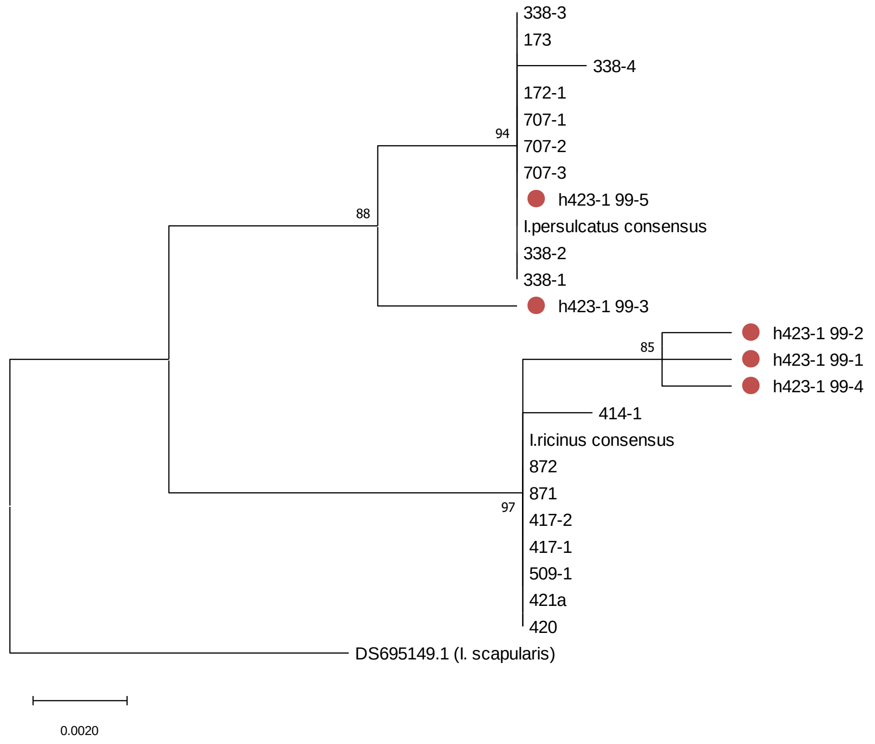

Figure S25. Maximum likelihood tree for the Toll_R gene fragments sequenced from molecular clones obtained from hybrid tick **h423-2**♂. Sequences from hybrid tick are marked with a red dot. The tree with the highest log likelihood (-1128.77) is shown after performing 1000 bootstrap replicates. The percentage of trees (> 70%) in which the associated taxa clustered together is shown next to the branches. The tree is drawn to scale, with branch lengths measured in the number of substitutions per site. Evolutionary analyses were conducted in MEGA X [22]

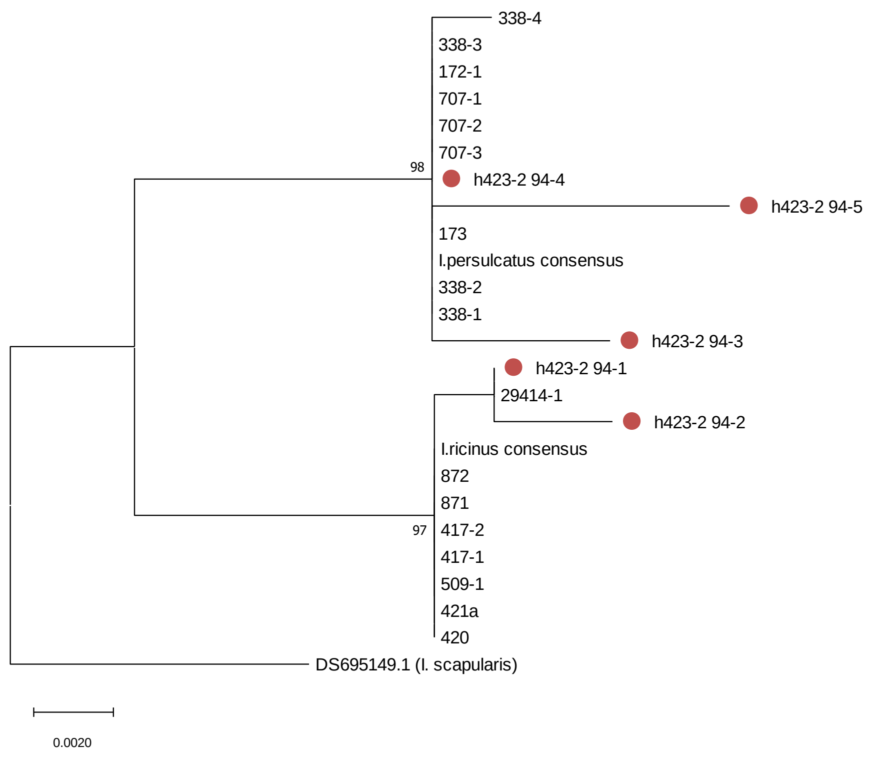

Supplement: Supplementary file 1 [file microorganisms-10-01306-s001.zip › microorganisms-1767970-supplementary.pdf]
